# Thymic myeloid cells are heterogenous and include a novel population of transitional dendritic cells

**DOI:** 10.1084/jem.20250733

**Published:** 2025-10-22

**Authors:** Matouš Vobořil, Fernando Bandeira Sulczewski, Ryan J. Martinez, K. Maude Ashby, Michael Manoharan Valerio, Juliana Idoyaga, Kristin A. Hogquist

**Affiliations:** 1 Center for Immunology, Department of Lab Medicine and Pathology, University of Minnesota Medical School, Minneapolis, MN, USA; 2 Department of Cell Biology, Faculty of Science, Charles University, Prague, Czech Republic; 3 Department of Pharmacology, University of San Diego School of Medicine, San Diego, CA, USA; 4 Department of Molecular Biology, University of California San Diego School of Biological Sciences, La Jolla, CA, USA

## Abstract

Myeloid cells, including dendritic cells (DCs) and macrophages, are essential for establishing central tolerance in the thymus by promoting T cell clonal deletion and regulatory T cell (Treg) generation. Previous studies suggest that the thymic DC pool consists of plasmacytoid DC (pDC), XCR1^+^ DC1, and SIRPα^+^ DC2. Yet the precise origin, development, and homeostasis, particularly of DC2, remain unresolved. Using single-cell transcriptomics and lineage-defining mouse models, we identify nine major populations of thymic myeloid cells and describe their lineage identities. What was previously considered to be “DC2” is actually composed of four distinct cell lineages. Among these are monocyte-derived DCs (moDCs) and monocyte-derived macrophages (moMacs), which are dependent on thymic IFN to upregulate MHCII and CD11c. We further demonstrate that conventional DC2 undergo intrathymic maturation through CD40 signaling. Finally, amongst DC2, we identify a novel thymic population of CX3CR1^+^ transitional DC (tDC), which represents transendothelial DCs positioned near thymic microvessels. Together, these findings reveal the thymus as a niche for diverse, developmentally distinct myeloid cells and elucidate their specific requirements for development and maturation.

## Teaser

Thymic myeloid cells regulate T cell tolerance. Here, we describe their heterogeneity. We found that thymic “DC2” comprise four distinct lineages, including monocyte-derived DCs and macrophages, and conventional DC2. Among DC2, a novel population of transitional DCs, representing thymus immigrating cells, was identified.

## Introduction

Thymic central tolerance is an essential process that protects the mammalian body against autoimmunity by forming a self-tolerant repertoire of T cells (Ashby and Hogquist, 2024). The scope of central tolerance is determined by the diversity of self-peptide–MHC complexes (self-p:MHC) that developing thymocytes recognize on thymic APCs. The thymus hosts various types of APCs, including thymic epithelial cells, and a diverse spectrum of APCs of hematopoietic origin, such as dendritic cells (DCs), macrophages, and B cells (Klein and Petrozziello, 2024). The role of thymic APCs of hematopoietic origin in central tolerance was first proposed in mice, where MHCII molecules were present only on radioresistant cells, but absent in hematopoietic lineage, which led to impaired negative selection of T cells and increased susceptibility to autoimmunity (van Meerwijk et al., 1997).

The thymic DC pool closely resembles the distribution of DCs in secondary lymphoid organs, including plasmacytoid DC (pDC), XCR1^+^ conventional DC type 1 (DC1), SIRPα^+^ DC2 (Li et al., 2009), as well as activated DCs (aDCs) counterparts, defined by CCR7 expression and increased MHC class II and co-stimulatory molecules expression (Ardouin et al., 2016). Unlike in peripheral tissues, DC activation in the thymus remains unchanged in germ-free mice, and it is not affected by genetic ablation of various innate immune signaling adaptors, indicating “sterile” forms of DC activation within the thymus (Ardouin et al., 2016; Oh et al., 2018). Recently, tonic expression of pro-inflammatory cytokines has been reported in the steady-state thymus, where they regulate the maturation and activation of various thymic DCs. For example, thymic CD301b^+^ DC2 require type II cytokine signaling (IL-4 and IL-13) for their maturation (Breed et al., 2022), while type III IFNs have been shown to regulate thymic DC1 and macrophage activation (Ashby et al., 2024). Additionally, CD40 signaling, along with the absence of single-positive (SP) thymocytes or MHC class II on hematopoietic cells results in a marked decrease in thymic aDC populations (Oh et al., 2018). Thus, tonic inflammatory signals, together with cognate interaction with SP thymocytes, promote the homeostatic activation of thymic DCs. However, the specific requirements for DC1 and DC2 maturation in the thymus remain poorly defined.

In the thymus, DC1 function is primarily associated with cross-presentation of medullary thymic epithelial cell (mTEC)-derived self-antigens to developing T cells facilitating the selection of regulatory T cells (Tregs) (Perry et al., 2014). Conversely, due to the high heterogeneity within SIRPα^+^ DC2, the specific function of DC2 in the thymus remains less well understood. Thymus-resident CD301b^+^ DC2 have been shown to be potent mediators of clonal deletion, as their genetic ablation alters the CD4SP thymocyte repertoire (Breed et al., 2022). Conversely, a subpopulation of SIRPα^+^ DC2 was described to originate in the periphery and thus be capable of presenting the antigens acquired in the periphery for the purpose of T cell selection (Bonasio et al., 2006). More recent research has identified the population of transendothelial DC positioned to the proximity of thymic microvessels, where they capture and present blood-borne antigens to developing T cells. The positioning of these cells depends on the CX3CR1-CX3CL1 axis (Vollmann et al., 2021). Furthermore, the population of CX3CR1^+^ DCs has been observed to increase in the thymus after the weaning period of mice, and these cells have been shown to be responsible for inducing the expansion of microbiota-specific T cells (Zegarra-Ruiz et al., 2021). This, along with newly described clusters of CX3CR1^+^ monocyte-derived DCs (moDCs) (Vobořil et al., 2020), as well as MHC class II^+^ CX3CR1^+^ macrophages (Zhou et al., 2022), present an extraordinary challenge in elucidating the developmental and functional heterogeneity within the thymic SIRPα^+^ DC2 population.

In this study, we used single-cell RNA sequencing (scRNA-seq) and various lineage-defining mouse models to investigate the origin, development, and homeostasis, particularly of thymic SIRPα^+^ cells. We show that the thymic SIRPα^+^ DC population includes populations of moDC and monocyte-derived macrophages (moMacs), defined by *Ms4a3*^*Cre*^ tracing and maintained through thymic IFN signaling. We further demonstrate that DC2 undergo intrathymic activation regulated by CD40 signaling to become CCR7^+^ aDC2. Finally, we identify a novel thymic population of transitional DC (tDC), sharing the developmental origin with pDC, that exhibit thymus-immigrating capacity and are positioned near thymic microvessels. Altogether, our study highlights the substantial heterogeneity within the SIRPα^+^ DC2 compartment and provides a developmental and functional characterization of individual thymic SIRPα^+^ DC subsets.

## Results

### scRNA-seq reveals heterogeneity in thymic DC2

**Figure S1.**
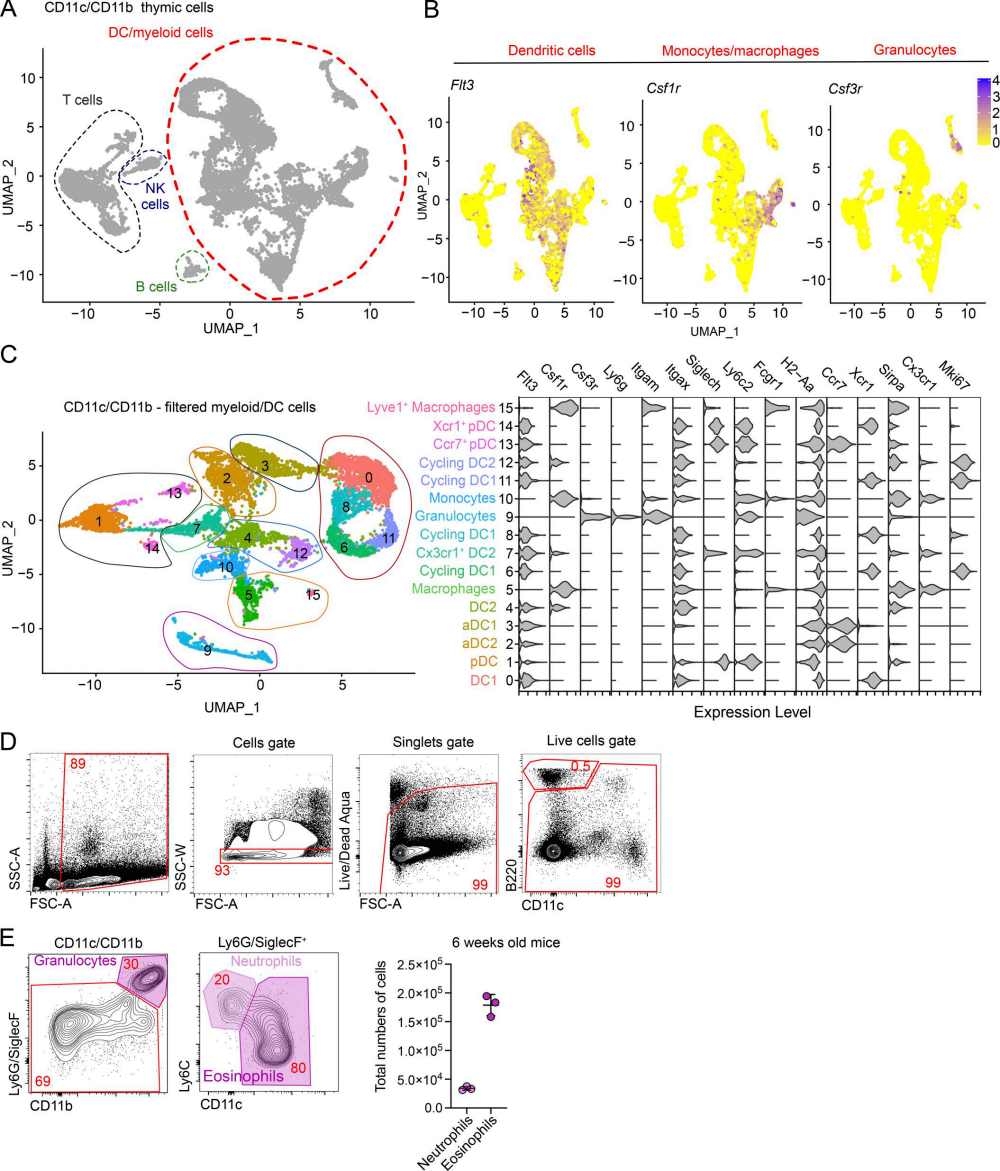
**scRNA-seq reveals heterogeneity in thymic DCs. (A)** scRNA-seq of CD11c^+^ and CD11b^+^ FACS-sorted cells from the thymus of 7-wk-old C57BL/6 mice. UMAP plots show the analysis of 11,586 transcriptome events, with dashed lines representing clusters expressing *Flt3*, *Csf1r*, and *Csf3r*. **(B)** Feature plots showing the normalized expression of *Flt3*, *Csf1r*, and *Csf3r* in the clusters defined in A. **(C)** UMAP plots show the analysis of 8,514 transcriptome events and identify 16 clusters of thymic myeloid cells. Violin plots show the normalized expression of signature genes in these clusters. **(D)** Representative flow cytometry gating strategy for pre-gating thymic myeloid cells. **(E)** Representative gating strategy for identifying thymic neutrophils (Ly6G/SiglecF^+^Ly6C^+^CD11c^−^) and eosinophils (Ly6G/SiglecF^+^Ly6C^−^CD11c^+^). The graph shows the total numbers of neutrophils and eosinophils per thymus in 7-wk-old C57BL/6 mice; *n* = 3 mice. Data are shown as mean ± SD.

**Figure 1.**
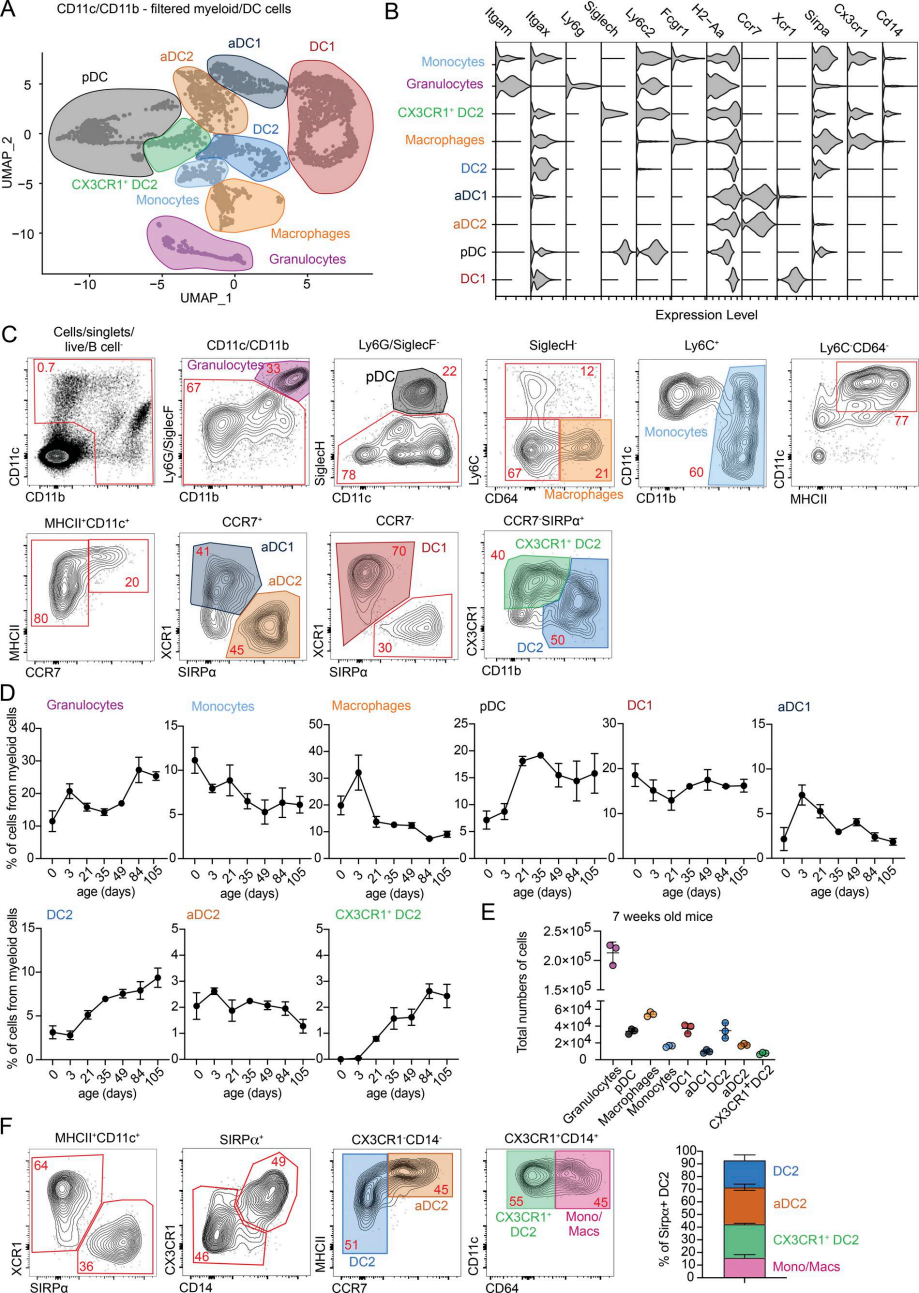
**scRNA-seq reveals heterogeneity in thymic DC2. (A)** scRNA-seq of CD11c^+^ and CD11b^+^ FACS-sorted cells from thymus of 7-wk-old C57BL/6 mice. Cells were bioinformatically filtered to include only clusters expressing *Flt3*, *Csf1r*, and *Csf3r*. UMAP plots show the analysis of 8,514 transcriptome events, identifying 9 major clusters, marked by color-coded lines. **(B)** Violin plots displaying normalized expression of signature genes associated with cell clusters defined in A. **(C)** Representative flow cytometry gating strategy for identifying the cell populations defined in A and B. Cells were pre-gated as shown in Fig. S1 D. The gating strategy identifies granulocytes (Ly6G/SiglecF^+^CD11b^+^), pDCs (SiglecH^+^), macrophages (Ly6C^−^CD64^+^), monocytes (Ly6C^+^CD11b^+^), aDC1 (CD11c^+^MHCII^+^CCR7^+^XCR1^+^), aDC2 (CD11c^+^MHCII^+^CCR7^+^SIRPα^+^), DC1 (CD11c^+^MHCII^+^ XCR1^+^), DC2 (CD11c^+^MHCII^+^SIRPα^+^CD11b^+^), and CX3CR1^+^ DC2 (CD11c^+^MHCII^+^SIRPα^+^CD11b^Low^CX3CR1^+^). **(D)** Frequency of thymic myeloid cell populations among total CD11c^+^ and CD11b^+^ cells in the thymus of C57BL/6 mice from birth (0 days old) to 105-day-old mice; *n* = 2–7 mice from two independent experiments. **(E)** Total numbers of cells per thymus in 7-wk-old C57BL/6 mice; *n* = 3 mice. **(F)** Representative gating strategy for thymic CD11c^+^MHCII^+^ SIRPα^+^ cells. The graph represents the percentage distribution of individual thymic subpopulations in 7-wk-old C57BL/6 mice; *n* = 2 mice from two independent experiments. Data are shown as mean ± SD.

To thoroughly characterize thymic DC/myeloid cell populations, we used scRNA-seq of sorted CD11c^+^ and CD11b^+^ cells from 7-wk-old C57BL/6 mice (Fig. S1 A) (Breed et al., 2022). Bioinformatically, we filtered the clusters expressing Fms-related receptor tyrosine kinase 3 (*Flt3*), and CSF 1 and 3 receptors (*Csf1r* and *Csf3r*) to identify DCs, monocyte/macrophages, and granulocytes (Fig. S1 B). After dimensionality reduction and re-clustering, we identified 16 cell clusters based on signature gene expression (Fig. S1 C). These clusters were assigned to nine major populations of thymic myeloid cells by their expression of lineage-defining genes (Fig. 1, A and B).

The thymus contains populations of granulocytes (*Csf3r*, *Itgam*, and *Ly6g*), monocytes (*Csf1r*, *Ly6c2*, and *Itgam*), and macrophages (*Csfr1* and *Fcgr1*). Notably, both monocytes and macrophages contain subpopulations of cells expressing higher levels of MHC class II (*H2-Aa*) and CD11c (*Itgax*), suggesting their activated phenotype (Ashby et al., 2024; Zhou et al., 2022) (Fig. 1, A and B; and Fig. S1 C). The thymic DC compartment includes pDC (*Siglech* and *Ly6c2*) and both immature and mature conventional DC—DC1 (*Xcr1*) and DC2 (*Sirpa*)—with the mature forms defined by the expression of *Ccr7* (Fig. 1, A and B). Proliferating “cycling” subclusters, marked by *Mki67* expression, are present in both DC1 and DC2 populations (Fig. S1 C). Interestingly, the analysis revealed a unique population of CX3CR1^+^ DC2 (in green), which clustered separately from conventional DC2 and aDC2. This population co-expressed some genes associated with pDC (*Siglech* and *Ly6c2*) as well as *Cd14* and *Sirpa* (Fig. 1, A and B).

We next established a flow cytometric panel enabling the discrimination of all nine major populations of thymic myeloid cells identified by scRNA-seq (Fig. 1, A and B). To identify these cells, we gated out B cells and focused on thymic cells expressing CD11c, CD11b, or both. Granulocytes (CD11b^+^ Ly6G/SiglecF^+^), pDC (SiglecH^+^), macrophages (CD64^+^Ly6C^−^), and monocytes (Ly6C^+^ CD11b^+^) were gated sequentially (Fig. S1 D and Fig. 1 C). Within the granulocyte population, we discriminated eosinophils (CD11c^+^ Ly6C^Low^) and neutrophils (CD11c^−^Ly6C^+^) (Fig. S1 E). Thymic DCs were defined as CD64^−^Ly6C^−^CD11c^+^MHCII^+^, and aDCs were discriminated as MHCII^High^ CCR7^+^. The expression of XCR1 and SIRPα was used to identify cells of DC1 and DC2 lineage, respectively. Notably, CX3CR1^+^ DC2 did not express the conventional DC1 marker XCR1 or the DC2/monocyte marker CD11b (Fig. 1 C).

To evaluate age-related changes in the thymic myeloid cell compartment, we applied the previously described gating strategy and defined the proportion of individual subsets within the CD11c/CD11b population from mice aged 0–105 days (15 wk). Consistent with prior findings, the DC1 and granulocyte populations maintained stable proportions from birth, whereas DC2 and pDC numbers increased during the first weeks of life (Fig. 1 D) (Breed et al., 2022). The aDC1, aDC2, and macrophage populations peaked around 3 days of life and subsequently decline with age. Interestingly, the CX3CR1^+^DC2 population was absent in the thymus before 21 days of age, suggesting these cells may correspond to previously described thymus-immigrating CX3CR1^+^ DCs (Zegarra-Ruiz et al., 2021) (Fig. 1 D). Numerically, by 7 wk of age, eosinophils were the most abundant thymic myeloid cells followed by macrophages (Fig. S1 E and Fig. 1 E). pDC, DC1, and DC2 constitute nearly equal fractions of thymic cells, while aDC1, aDC2, monocytes, and CX3CR1^+^ DC2 remained rare (Fig. 1 E).

Based on the above data, the population of thymic MHCII^+^CD11c^+^SIRPα^+^ cells, originally identified as conventional DC2, shows much higher internal heterogeneity (Fig. 1, B and C). Previously, it was described that SIRPα^+^ DCs contain a population of moDCs defined by the expression of CX3CR1, CD14, and Ly6C (Vobořil et al., 2020). Here, transcriptional data suggest that, in addition to moDCs, thymic SIRPα^+^ DCs also contain a distinct subpopulation of CX3CR1^+^ DC2 that cluster separately from monocytes and macrophages (Fig. 1, A and B). Flow cytometry analysis confirmed this, showing that thymic SIRPα^+^ DCs contain four subsets: conventional DC2, aDC2, CX3CR1^+^ DC2, and CD64^+^ monocyte/macrophage-derived cells, with all populations present in roughly similar proportion (Fig. 1 F).

### The thymus contains IFN-activated populations of moDC and moMacs

As described above, the thymus contains a primary population of monocytes (Ly6C^+^CD11b^+^) and macrophages (CD64^+^), of which some upregulate CD11c, MHCII, and SIRPα, which are classic markers of conventional DC2 (Fig. 1, B and C). Thus, cells with monocyte and macrophage markers constitute ∼15% of the thymic conventional DC2 gate (Fig. 1 F). Therefore, accurately distinguishing thymic monocyte/macrophage populations is essential for understanding the true heterogeneity of DC2s. Thymic macrophages are known to consist of two major subpopulations, defined by the expression of *Timd4* and *Cx3cr1* (Zhou et al., 2022), and depend on transcriptional factor *Nr4a1* (Tacke et al., 2015). It has been reported that *Timd4*^*+*^ macrophages are of embryonic origin, while *Cx3cr1*^*+*^ macrophages are derived from adult hematopoietic stem cells (Zhou et al., 2022).

**Figure 2.**
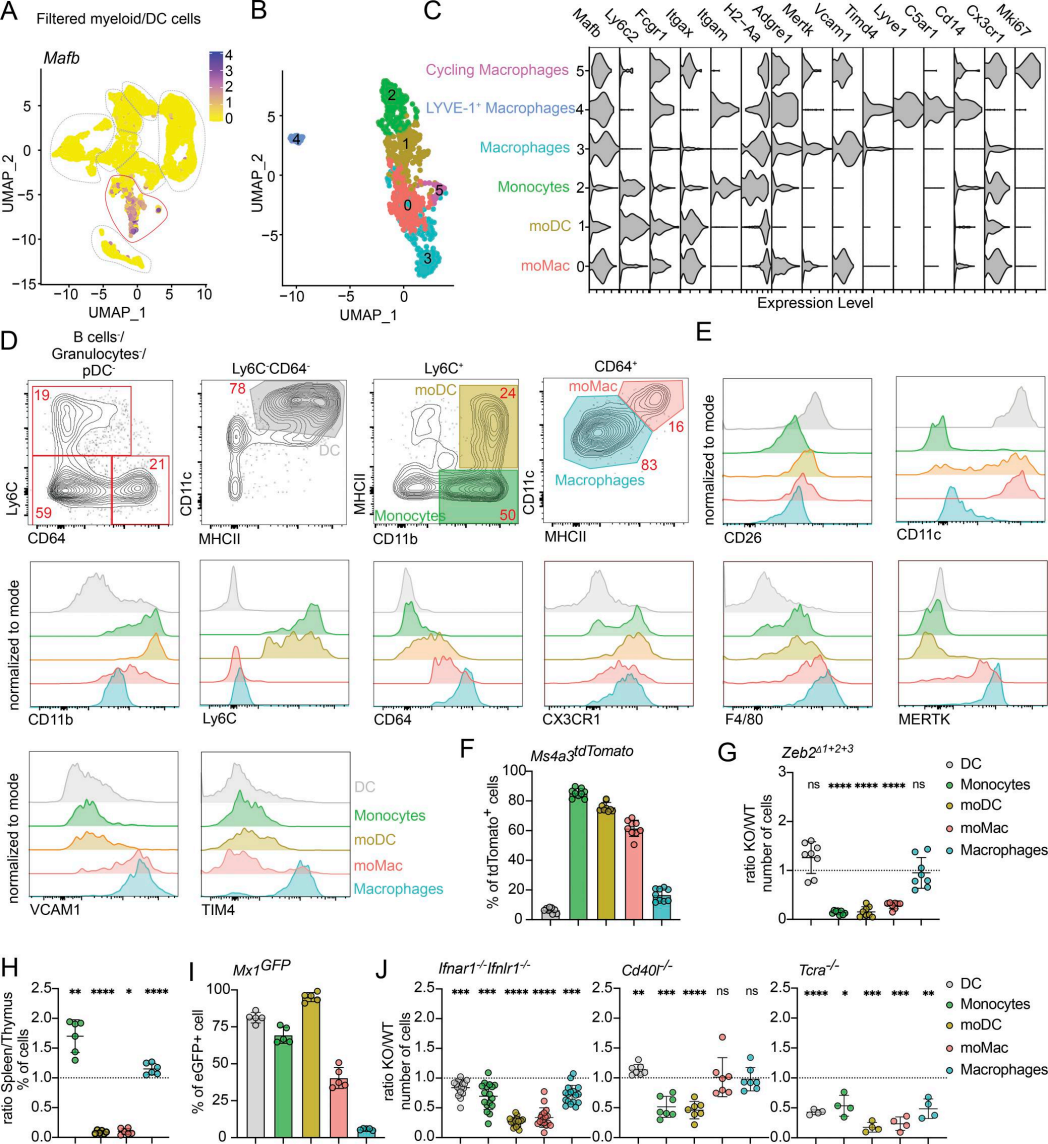
**The thymus contains IFN-activated populations of moDC and moMacs. (A)** Feature plot showing the normalized expression of *Mafb* in clusters identified in Fig. 1 A. **(B)** scRNA-seq of CD11c^+^ and CD11b^+^ FACS-sorted cells from the thymus of 7-wk-old C57BL/6 mice. Cells were bioinformatically filtered to include only clusters expressing *Mafb*. UMAP plots show the analysis of 1,020 transcriptome events, identifying five clusters. **(C)** Violin plots displaying the normalized expression of signature genes associated with cell clusters defined in B. **(D)** Representative flow cytometry gating strategy for identifying the four major populations defined in B and C. The gating strategy identifies monocytes (Ly6C^+^CD11b^+^MHCII^−^), moDC (Ly6C^+^CD11b^+^MHCII^+^), macrophages (CD64^+^CD11c^−^MHCII^−^), and moMacs (CD64^+^CD11c^+^MHCII^+^). **(E)** Representative flow cytometry plots showing normalized expression of CD26, CD11c, CD11b, LY6C, CD64, CX3CR1, F4/80, MERTK, VCAM1, and TIM4 in thymic DCs (Ly6C^−^CD64^−^CD11c^+^MHCII^+^) and thymic monocyte and macrophage populations described in D. **(F)** Frequency of tdTomato^+^ thymic cells (gated as in D) in *Ms4a3*^*Cre*^ ROSA26^tdTomato^ (*Ms4a3*^*tdTomato*^) mice; *n* = 9 mice from three independent experiments. **(G)** Numbers of thymic DC, monocyte, and macrophage (gated as in D) populations in *Zeb2*^*Δ1+2+3*^ mice, shown as KO/WT ratio of cell numbers; *n* = 8 mice from three independent experiments. **(H)** Numbers of thymic and splenic cells (gated as in D), shown as spleen/thymus ratio of cell frequencies; *n* = 6 mice from three independent experiments. **(I)** Frequency of eGFP^+^ thymic cells (gated as in D) in *Mx1*^*eGFP*^ mice; *n* = 5 mice from two independent experiments. **(J)** Numbers of thymic cells (gated as in D) in *Ifnar1*^*−/−*^*Ifnlr1*^*−/−*^, *Cd40l*^*−/−*^, and *Tcra*^*−/−*^ mice, shown as KO/WT ratio of cell numbers; *n* = 4–17 mice from at least two independent experiments. Data are shown as mean ± SD. Statistical analysis was performed by a one-sample *t* test and Wilcoxon test with a theoretical mean of 1, *P ≤ 0.05, **P ≤ 0.01, ***P ≤ 0.001, ****P ≤ 0.0001, and n.s., not significant.

To comprehensively analyze the heterogeneity in thymic monocyte/macrophage populations, we bioinformatically filtered cells expressing the transcription factor *Mafb*, which distinguishes monocytes and macrophages from other immune lineages (Wu et al., 2016) (Fig. 2 A). Re-clustering the data using only *Mafb*^+^ cells revealed five major subpopulations of thymic monocytes and macrophages. These included classical monocytes (*Ly6c2*, *Itgam*, and *Cx3cr1*), classical macrophages (*Fcgr1*, *Adgre1*, *Mertk*, and *Timd4*), and a population we will refer to as “moDC” (*Ly6c2*, *Fcgr1*, *Cx3cr1*, *H2-Aa*, and *Itgax*) and “moMacs,” defined by the expression of macrophage markers (*Adgre1*, *Mertk*, and *Vcam1*). The term moDC was used to reflect both the origin and the phenotypic and functional properties of these cells, which resemble conventional DCs (Guilliams et al., 2014). Consistent with previous findings (Zhou et al., 2022), thymic moMac exhibited a subset of proliferating cells, which we refer to as the *Mki67*^+^ cycling macrophage subpopulation. Finally, the thymus also harbors a small population of LYVE-1^+^ macrophages, characterized by the expression of *Lyve1* and *C5ar1* (CD88) (Fig. 2, B and C).

**Figure S2.**
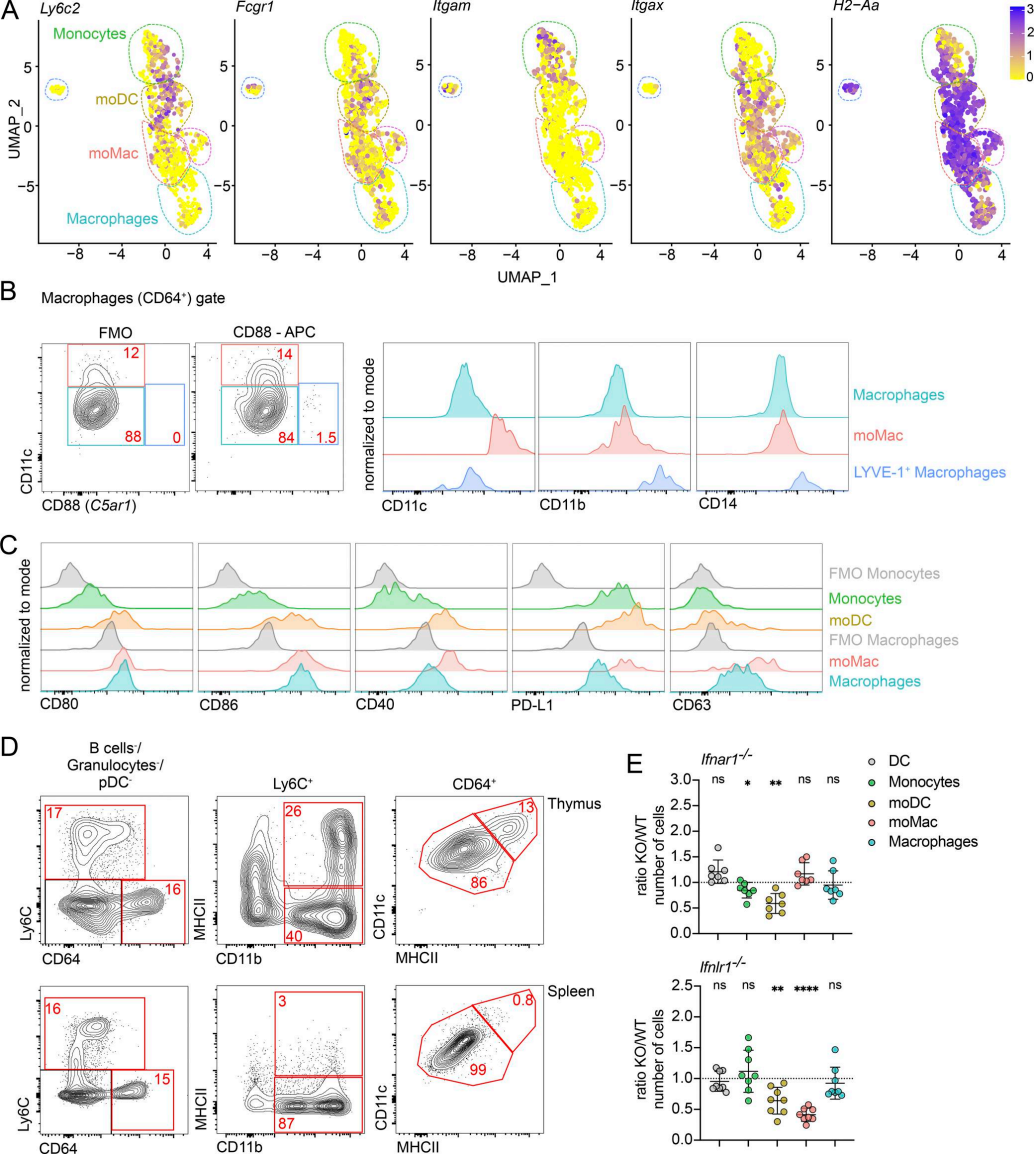
**The thymus contains IFN-activated populations of moDC and moMacs. (A)** Feature plot showing the normalized expression of *Ly6c2*, *Fcgr1*, *Itgam*, *Itgax*, and *H2-Aa* in clusters identified in Fig. 2 B. **(B)** Representative flow cytometry plots identifying LYVE-1^+^ macrophages using CD88, CD11c, CD11b, and CD14 antibody staining. **(C)** Representative flow cytometry plots showing the normalized expression of CD80, CD86, CD40, PD-L1, and CD63 in thymic DCs (Ly6C^−^CD64^−^CD11c^+^MHCII^+^) and thymic monocyte and macrophage populations described in Fig. 2 D. **(D)** Representative flow cytometry plots comparing the monocyte and macrophage populations between thymus and spleen. **(E)** Numbers of thymic cells (gated as in Fig. 2 D) in *Ifnar1*^*−/−*^ and *Ifnlr1*^*−/−*^ mice, shown as KO/WT ratio of cell numbers; *n* = 7–8 mice from at least two independent experiments. Data are shown as mean ± SD. Statistical analysis was performed by a one-sample *t* test and Wilcoxon test with a theoretical mean of 1; *P ≤ 0.05, **P ≤ 0.01, ****P ≤ 0.0001, and n.s., not significant.

Using transcriptional data (Fig. S2 A), we designed a flow cytometry panel to define the major populations: monocytes (Ly6C^+^CD11b^+^MHCII^−^), macrophages (Ly6C^−^CD64^+^MHCII^Low^CD11c^Low^), moDC (Ly6C^+^CD11b^+^MHCII^+^), and moMac (Ly6C^−^CD64^+^MHCII^High^CD11c^High^) (Fig. 2 D) Notably, moDC also expressed CD64, although their expression levels were lower than in moMac or classical macrophages (Fig. 1 F and Fig. 2 E). We also verified the presence of LYVE-1^+^ macrophages in the thymus, using CD88 staining and testing additional markers. These cells are very rare, as they constitute ∼1.5% of all thymic macrophages (Fig. S2 B). Consistent with their expression of myeloid lineage restricted *Mafb*, these populations expressed only very low levels of DC-defining molecules CD26 or *Flt3*, suggesting minimal DC contamination in the gating strategy (Fig. 2 E and Fig. S1 B). To validate our gating strategy, we tested the expression of several monocyte/macrophage prototypical markers via flow cytometry. Our results indicate that the macrophage population corresponds to the previously described *Timd4*^+^ macrophages (Zhou et al., 2022), as these cells upregulate MERTK and TIM4 while exhibiting lower CD11b expression (Fig. 2 E). Despite the transcriptional similarities between moDCs and moMacs, the upregulation of macrophage-specific markers (MERTK and VCAM1) in moMacs further supports their classification as part of the macrophage lineage (Fig. 2 E).

Although conventional DC2 and moDC/moMac exhibit distinct transcriptional profiles, establishing the lineage origin of these cells based on surface markers alone remains challenging. To verify the monocyte origin of moDC and moMac, we utilized the *Ms4a3*^*Cre*^ ROSA26^tdTomato^ (*Ms4a3*^*tdTomato*^) fate-mapping system. The *Ms4a3* gene is specifically expressed in granulocyte-monocyte progenitors, but not in monocyte-DC progenitors (MDPs) or subsequent DC progeny, enabling clear tracking of monocyte-derived cells (Liu et al., 2019). Our data indicate high levels of recombination within the monocyte, moDC, and moMac populations, strongly supporting their monocyte origin (Fig. 2 F). Conversely, the conventional DC population showed minimal recombination, confirming the very limited contamination by DCs in our gating strategy. Interestingly, the macrophage population exhibited low recombination levels as well, suggesting that these cells predominantly represent embryonically derived tissue-resident macrophages (Fig. 2 F) (Liu et al., 2019). These findings were corroborated by using mice carrying triple mutations in the *Zeb2* enhancer (*Zeb2*^*Δ1+2+3*^), which results in the absence of conventional DC2 and monocytes but not tissue-resident macrophages (Liu et al., 2022). In this system, we observed the depletion of monocytes, moDC, and moMacs, while macrophages remained unaffected (Fig. 2 G).

Both moDCs and moMacs resemble fully matured APC populations, characterized by high expression of MHCII and various co-stimulatory molecules (Fig. S2 C). To explore their distribution in peripheral tissues, we compared these populations in the thymus and spleen. Surprisingly, moDCs and moMacs were almost entirely absent in the steady-state spleen, unlike in the thymus (Fig. 2 H and Fig. S2 D), suggesting that their maturation occurs intrathymically. Previously, it was reported that most thymic APCs respond to sterile IFN at steady state, evidenced by upregulating *Mx1* (Ashby et al., 2024). We further tested monocytes and macrophages for their ability to respond to thymic IFN and examined their dependency on IFN signaling. The analysis of *Mx1*^*eGFP*^ mice confirmed that monocytes, moDC, and 50% of moMac express GFP, whereas classical macrophages do not (Fig. 2 I). Consistent with this, moDC and moMac maturation depended on thymic IFNs, as mice lacking type I and III IFN receptors (*Ifnar1*^*−/−*^*Ifnlr1*^*−/−*^) exhibited a significant reduction in these populations (Fig. 2 J). Notably, while moMac maturation depended largely on type III IFNs, moDC maturation depended on both types of IFNs (Fig. S2 E). Given the role of CD40 signaling and SP thymocytes in thymic DC maturation (Oh et al., 2018; Spidale et al., 2014), we tested their involvement in monocyte and macrophage maturation. Interestingly, CD40 signaling appeared to play a minimal role, as *Cd40l*^*−/−*^ mice showed only a minor reduction in total monocyte and moDC numbers, with no specific impact on moMacs. Conversely, the absence of SP thymocytes in *Tcra*^*−/−*^ mice significantly reduced DC, moDC, and moMac populations, while other cell types, such as granulocytes and pDC, remained unaffected (Fig. 2 J). This finding highlights the key role of SP thymocytes in monocyte maturation through driving IFN production in thymic epithelial cells (TECs).

Together, the thymus contains populations of monocytes and macrophages, along with moDCs and moMacs, whose maturation relies on IFN signaling and the presence of SP thymocytes.

### Activation of conventional DC1 and DC2 requires distinct signals

**Figure S3.**
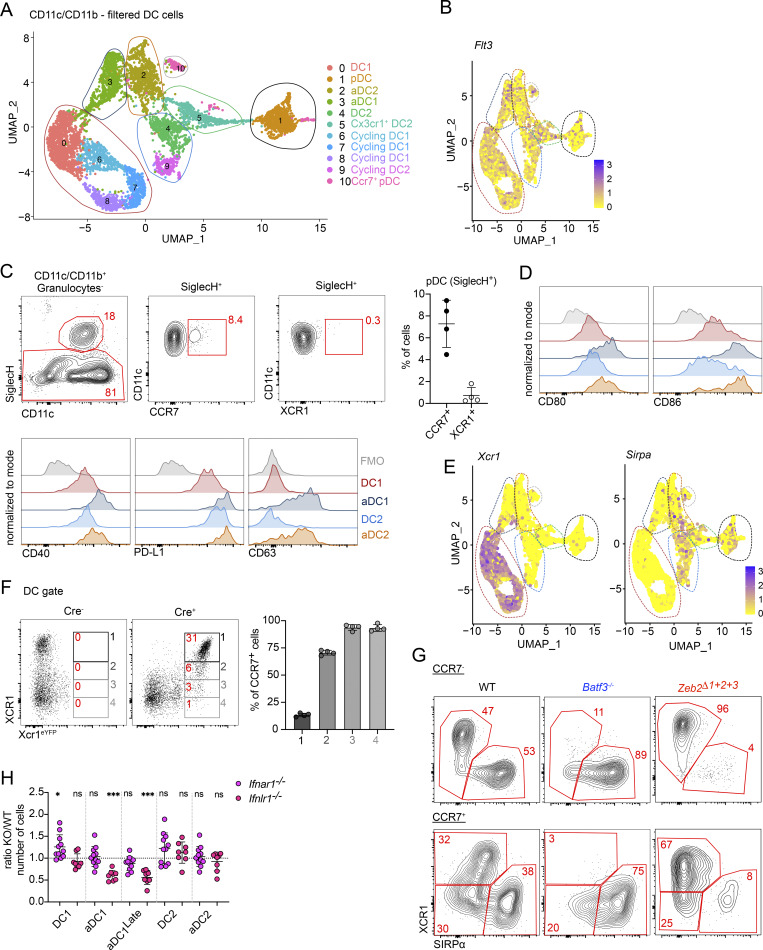
**Activation of conventional DC1 and DC2 requires distinct signals. (A)** scRNA-seq of CD11c^+^ and CD11b^+^ FACS-sorted cells from the thymus of 7-wk-old C57BL/6 mice. Cells were bioinformatically filtered to include only clusters expressing *Flt3*. UMAP plots show the analysis of 6,928 transcriptome events, identifying 11 clusters. **(B)** Feature plot showing the normalized expression of *Flt3* in clusters identified in Fig. 3 A. **(C)** Representative flow cytometry plots identifying CCR7^+^ and XCR1^+^ pDCs in the thymus. The graph shows frequency of CCR7^+^ and XCR1^+^ cells within the thymic SiglecH^+^ population; *n* = 4 mice. **(D)** Representative flow cytometry plots showing the normalized expression of CD80, CD86, CD40, PD-L1, and CD63 in thymic DC populations defined in Fig. 3 B. **(E)** Feature plots showing the normalized expression of *Xcr1* and *Sirpa* in clusters identified in Fig. 3 A. **(F)** Representative flow cytometry plots showing expression of XCR1 in eYFP^+^ cells from *Xcr1*^*iCre*^*Rosa26*^*eYFP*^ (*Xcr1*^*eYFP*^) mice. The graph shows frequency of CCR7^+^ cells in cell populations defined by flow cytometry; *n* = 4 mice, from two independent experiments. **(G)** Representative flow cytometry plots comparing the thymic DCs (gated as in Fig. 3 C) in *Batf3*^*−/−*^ and *Zeb2*^*Δ1+2+3*^ mice. **(H)** Numbers of thymic DCs (gated as in Fig. 3 C) in *Ifnar1*^*−/−*^ and *Ifnlr1*^*−/−*^ mice, shown as KO/WT ratio of cell numbers; *n* = 8–11 mice from at least three independent experiments. Data are shown as mean ± SD. Statistical analysis was performed by a one-sample *t* test and Wilcoxon test with a theoretical mean of 1, *P ≤ 0.05, ***P ≤ 0.001, and n.s., not significant.

**Figure 3.**
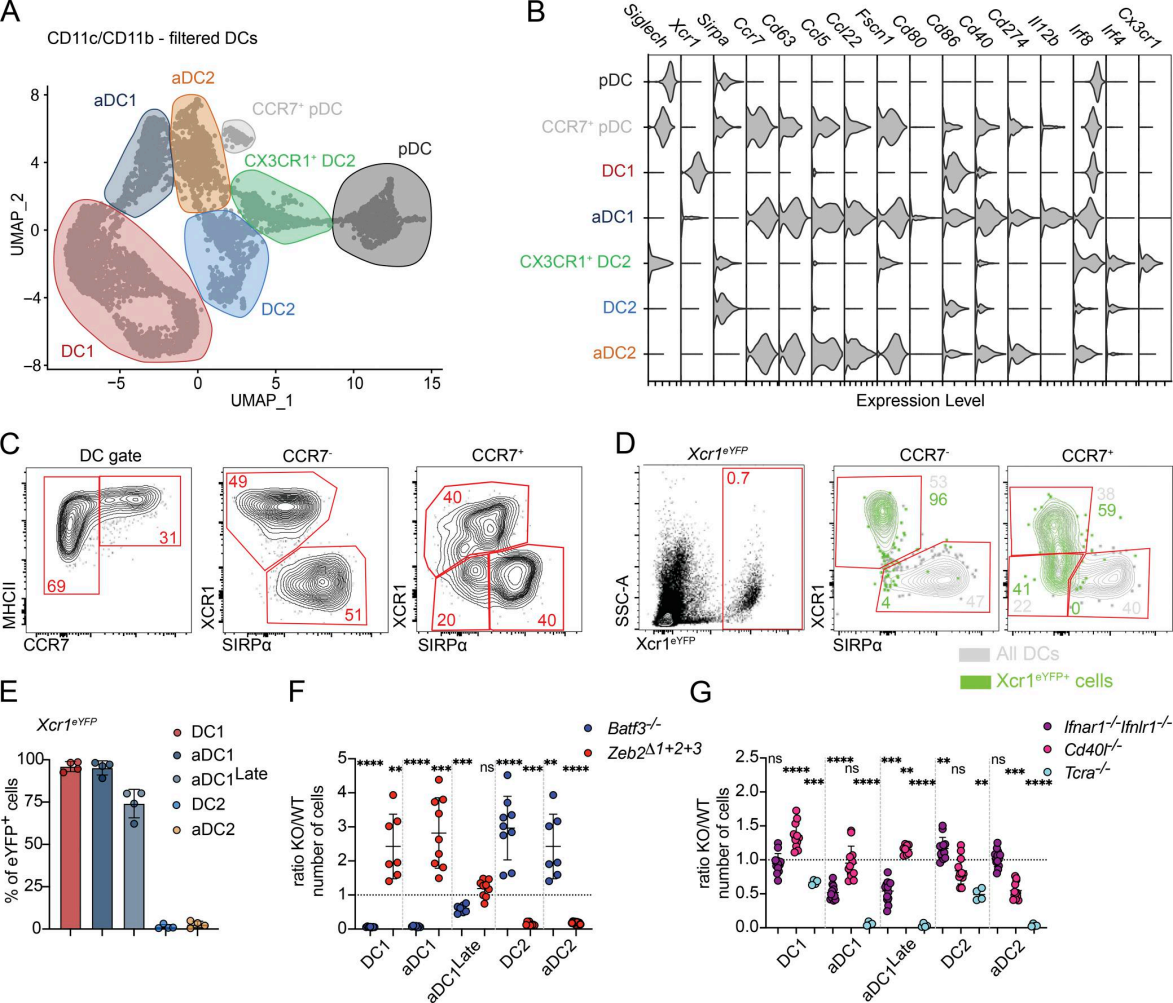
**Activation of conventional DC1 and DC2 requires distinct signals. (A)** scRNA-seq of CD11c^+^ and CD11b^+^ FACS-sorted cells from the thymus of 7-wk-old C57BL/6 mice. Cells were bioinformatically filtered to include only clusters expressing *Flt3*. UMAP plots show the analysis of 6,928 transcriptome events, identifying seven major clusters, marked by color-coded lines. **(B)** Violin plots displaying the normalized expression of signature genes associated with cell clusters defined in A. **(C)** Representative flow cytometry gating strategy for identifying thymic DCs (Ly6C^−^CD64^−^CD11c^+^MHCII^+^), DC1 (CCR7^−^XCR1^+^), aDC1 (CCR7^+^XCR1^+^), DC2 (CCR7^−^SIRPα^+^), and aDC2 (CCR7^+^SIRPα^+^). **(D)** Representative flow cytometry plots showing expression of eYFP by thymic DC populations described in C in *Xcr1*^*iCre*^*Rosa26*^*eYFP*^ (*Xcr1*^*eYFP*^) mice. Gray cells represent all thymic cells gated as in C; green cells represent eYFP^+^ cells. **(E)** Frequency of eYFP^+^ cells (gated as in C) in *Xcr1*^*iCre*^*Rosa26*^*eYFP*^ (*Xcr1*^*eYFP*^) mice; *n* = 4 mice from three independent experiments. **(F)** Numbers of thymic DCs (gated as in C) in *Batf3*^*−/−*^ and *Zeb2*^*Δ1+2+3*^ mice, shown as KO/WT ratio of cell numbers; *n* = 7–9 mice from three independent experiments. **(G)** Numbers of thymic DCs (gated as in C) in *Ifnar1*^*−/−*^*Ifnlr1*^*−/−*^, *Cd40l*^*−/−*^, and *Tcra*^*−/−*^ mice, shown as KO/WT ratio of cell numbers; *n* = 4–13 mice from at least two independent experiments. Data are shown as mean ± SD. Statistical analysis was performed by a one-sample *t* test and Wilcoxon test with a theoretical mean of 1; **P ≤ 0.01, ***P ≤ 0.001, ****P ≤ 0.0001, and n.s., not significant.

After characterizing thymic populations of moDCs and moMacs, we focused on the heterogenous populations of thymic DCs. To distinguish DCs from other lineages, we bioinformatically filtered cells expressing *Flt3*. Re-clustering the data revealed 11 subclusters of *Flt3*^+^ cells (Fig. S3, A and B), which we further classified into 7 major thymic DC clusters (Fig. 3, A and B). Within these, we annotated the populations of pDCs, DC1, and DC2, all of which included cycling *Mki67*-expression cells, as well as a distinct subset of CX3CR1^+^ DC2 (Fig. 3 A an Fig. S3 A).

We identified three populations of *Ccr7*^+^ cells corresponding to the previously defined mature population of aDCs. According to an earlier study, aDC1 have undergone homeostatic maturation within the thymus, characterized by the overexpression of maturation-related genes, including chemokines, cytokines, and co-stimulatory molecules (*Ccr7*, *Ccl5*, *Ccl22*, *Fscn1*, *Cd40*, and *Il12b*) (Ardouin et al., 2016). The expression of these genes was significantly enriched in the cluster annotated as aDC1, confirming their fully activated phenotype (Fig. 3 B). Interestingly, we also detected the expression of these maturation-related genes in a population of CCR7^+^ DC2 and CCR7^+^ pDC, suggesting they may represent thymus-specific subsets of homeostatically aDC2 and pDC, respectively (Fig. 3, A and B). Flow cytometry analysis further confirmed the expression of CCR7 on thymic DC2 and pDC, confirming their activated state (Fig. 3 C and Fig. S3 C). Conversely, XCR1 staining did not validate the presence of *Xcr1*^*+*^ pDC identified through scRNA-seq analysis, so these cells were excluded from analysis (Fig. S1 C and Fig. S3 C). After gating out pDC, antibody staining of MHCII^High^ CCR7^+^ DCs distinguishes the total aDC population, with XCR1 and SIPRα protein staining identifying aDC1 and aDC2, respectively (Fig. 3 C). The expression of co-stimulatory molecules CD80, CD86, CD40, PD-L1, and CD63 clearly confirms the activated phenotype of both populations (Fig. S3 D). Notably, the protein expression of XCR1 in CCR7^+^ populations is lower than in their immature CCR7^−^ counterpart (Fig. 3 C). This corresponds to the almost negligible mRNA expression of *Xcr1* in these aDCs (Fig. S3 E). Furthermore, the flow cytometry analysis of MHCII^High^ CCR7^+^ reveals a subpopulation of XCR1^−^SIPRα^−^ double-negative cells, whose lineage-specific origin we wanted to determine (Fig. 3 C).

To determine the origin of double-negative aDCs, we used the *Xcr1*^*iCre*^*Rosa26*^*eYFP*^ DC1 lineage-tracing mouse model, tracking the history of *Xcr1* expression (Ferris et al., 2020). As expected, eYFP expression was restricted to DC1 and aDC1 cells but was also highly enriched in XCR1^−^SIPRα^−^ double-negative aDCs, with ∼75% of these cells expressing eYFP despite the complete absence of XCR1 protein expression (Fig. 3, D and E). Moreover, eYFP^+^ cells with low or negligible XCR1 expression showed an increased proportion of CCR7^+^ cells, suggesting that DC1 downregulates XCR1 upon activation while acquiring the CCR7^+^ aDC phenotype (Fig. S3 F). Based on this, XCR1^−^SIPRα^−^ double-negative aDCs likely represent a later stage of DC1 activation (aDC1^Late^), corresponding to the previously identified human aDC3 cells (Park et al., 2020).

Whereas the origin of aDC1 has been attributed to DC1 lineage (Ardouin et al., 2016), the ontogeny of aDC2 has not yet been reported. To investigate this further, we used two lineage-depleting mouse models: *Batf3*^*−/−*^ mice, which lack DC1 cells (Hildner et al., 2008), and mice carrying triple mutations in the *Zeb2* enhancer (*Zeb2*^*Δ1+2+3*^), which lack DC2 lineage (Liu et al., 2022). Analysis of thymic CCR7^+^ aDCs from these mice showed that both XCR1^+^ aDC1 and XCR1^−^ aDC1^Late^ cells were significantly reduced only in *Batf3*^*−/−*^ mice, whereas SIRPα^+^ aDC2 were depleted in *Zeb2*^*Δ1+2+3*^ mice (Fig. 3 F and Fig. S3 G). This clearly confirms that despite their extensive transcriptional similarities, aDC1 and aDC2 originate from distinct precursors. Interestingly, the total number of thymic DCs remains unchanged in both *Batf3*^*−/−*^ and *Zeb2*^*Δ1+2+3*^, as the depletion of one DC subset is compensated by an increase in the other subset (Fig. 3 F).

The specific requirements for DC1 and DC2 maturation in the thymus remain poorly defined. Previous studies suggested that the maturation of both thymic DC1 and DC2 is markedly reduced in mice lacking CD40 signaling, as well as in those lacking SP thymocytes (Oh et al., 2018; Spidale et al., 2014). More recently, type I and type III IFNs were shown to regulate maturation of DC1 but not DC2, despite both populations expressing IFN receptors (Ashby et al., 2024). We applied the previously described gating strategy to verify the dependence of aDC1 populations, as well as aDC2, on IFNs. As expected, the numbers of aDC1 and aDC1^Late^ cells were significantly reduced in *Ifnar1*^*−/−*^*Ifnlr1*^*−/−*^ mice, whereas aDC2 remained unchanged (Fig. 3 G). As previously described, this reduction was primarily associated with diminished type III IFN signaling (Fig. S3 H). Conversely, mice deficient for CD40-ligand (*Cd40l*^*−/−*^) showed a significant reduction in aDC2 numbers, but in contrast to a previous publications (Oh et al., 2018; Spidale et al., 2014), no decrease in either aDC1 or aDC1^Late^ cells (Fig. 3 G). These findings indicate that despite their transcriptional similarities, aDC1 and aDC2 require distinct signals for thymic maturation. Moreover, *Tcra*^*−/−*^ mice showed significantly altered activation of both DC1 and DC2 populations, as previously reported (Oh et al., 2018; Spidale et al., 2014), highlighting the role of SP thymocytes in DC maturation—either by providing CD40L or driving IFN production (Fig. 3 G).

Together, these findings demonstrate that the thymus harbors fully activated subsets of DC1 and DC2, whose maturation depends on IFN signaling and CD40L signaling, respectively.

### The thymus contains a population of CX3CR1^+^ tDC

**Figure 4.**
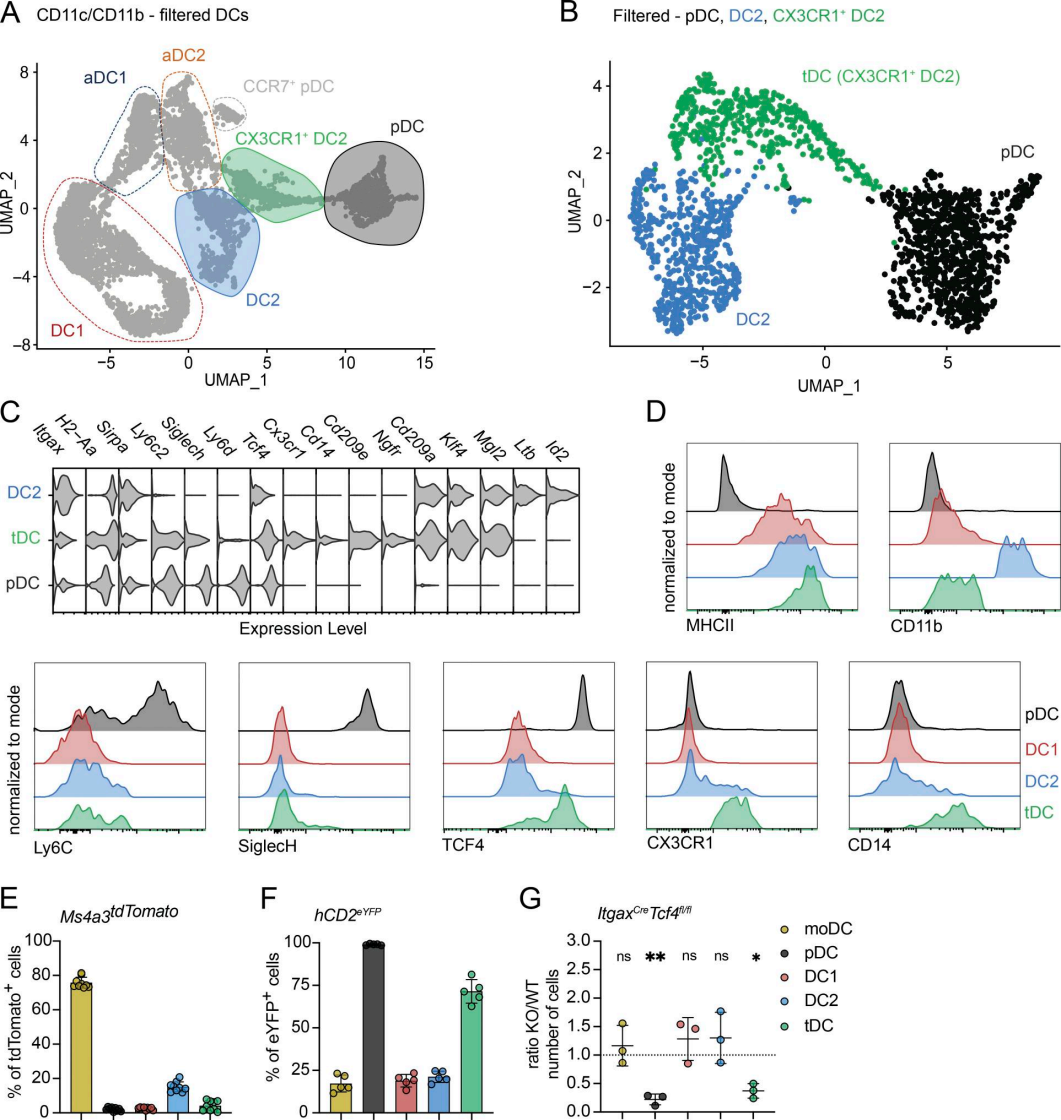
**The thymus contains a population of CX3CR1** ^**+**^ **tDC. (A)** scRNA-seq of CD11c^+^ and CD11b^+^ FACS-sorted cells from the thymus of 7-wk-old C57BL/6 mice. Cells were bioinformatically filtered and displayed as described in Fig. 3 A. DC2, CX3CR1^+^ DC2, and pDC are marked by color-coded lines. **(B)** UMAP plots showing the distribution of filtered DC2, CX3CR1^+^ DC2, and pDC thymic populations defined in A. **(C)** Violin plots displaying the normalized expression of signature genes associated with cell clusters defined in B. **(D)** Representative flow cytometry plots showing the normalized expression of MHCII, CD11b, Ly6C, SiglecH, TCF4, CX3CR1, and CD14 in thymic DC populations defined in B and gated according to the Fig. S4 A. **(E)** Frequency of tdTomato^+^ thymic DC populations (gated as in Fig. S4 A) in *Ms4a3*^*Cre*^ ROSA26^tdTomato^ (*Ms4a3*^*tdTomato*^) mice; *n* = 9 mice from three independent experiments, and moDC are used as control. **(F)** Frequency of eYFP^+^ thymic DC populations (gated as in Fig. S4 A) in *hCD2*^*iCre*^ ROSA26^eYFP^ (*hCD2*^*eYFP*^) mice; *n* = 5 mice from three independent experiments. **(G)** Numbers of thymic populations (gated as in Fig. S4 A) in *Itgax*^*Cre*^*Tcf4*^*fl/fl*^ mice, shown as KO/WT ratio of cell numbers; *n* = 3 mice from two independent experiments, and *Itgax*^*Cre−*^ mice were used as controls. Data are shown as mean ± SD. Statistical analysis was performed by a one-sample *t* test and Wilcoxon test with a theoretical mean of 1; *P ≤ 0.05, **P ≤ 0.01, and n.s., not significant.

Apart from moDC and moMac, thymic CX3CR1^+^SIRPα^+^ DCs also include a distinct subpopulation of CX3CR1^+^ DC2, which clusters separately from conventional DC2 and aDC2 (in green, Fig. 1, A and F). Based on their transcriptional profile, CX3CR1^+^ cells share similarity to DC2 but also to thymic pDCs (Fig. 3, A and B). To investigate this further, we compared the transcriptional profiles of thymic DC2, CX3CR1^+^ DC2, and pDCs by bioinformatically filtering and re-clustering these populations (Fig. 4, A and B). This analysis revealed that CX3CR1^+^ DC2 expresses some genes characteristic of pDCs—such as *Ly6c2*, *Siglech*, *Ly6d*, and *Tcf4*—as well as some characteristic of DC2, including *Irf4*, *Cd209a*, *Klf4*, and *Mgl2*. Additionally, we identified a set of genes uniquely expressed in the CX3CR1^+^ DC2 population, such as *Cd209e*, *Ngfr*, *Cx3cr1*, and *Cd14* (Fig. 4 C). Flow cytometric analysis confirmed the expression of TCF4, CX3CR1, and CD14 at the protein level, defining this population as TCF4^+^CX3CR1^+^CD14^+^ and SiglecH^−^CD11b^Low^ (Fig. 4 D).

This pattern suggested to us that thymic CX3CR1^+^ DC2 might represent a population called tDCs, recently described in peripheral tissues (Leylek et al., 2019; Sulczewski et al., 2023; Rodrigues et al., 2023). tDCs were initially identified in human blood and have been shown to be conserved between mice and humans (Alcántara-Hernández et al., 2017; Leylek et al., 2019; Villani et al., 2017). The term “tDC” was used to highlight their transcriptomics, phenotypic, and functional features, which span characteristics of both pDCs and DC2 (Leylek et al., 2019). Therefore, we decided to employ lineage-marking and fate-mapping approaches to test if thymic “CX3CR1^+^ DC2” are equivalent to peripheral tDC.

Phenotypically, CX3CR1^+^ DC2 resemble monocyte-derived cells by the expression of CX3CR1 and CD14; however, tDC are not of monocyte origin but instead share a developmental lineage with pDCs (Sulczewski et al., 2023; Rodrigues et al., 2024). To investigate the origin of CX3CR1^+^ DC2, we assessed their recombination levels in the monocyte lineage-tracer *Ms4a3*^*Cre*^ ROSA26^tdTomato^ mice. Indeed, CX3CR1^+^ DC2 were not marked by tdTomato in these mice (Fig. 4 E), indicating that they are not a monocyte-derived population. Alternatively, we utilized a pDC-specific lineage-tracing model, expressing iCre under the human CD2 promoter (*hCD2*^*iCre*^), crossed with *ROSA26*^*eYFP*^ mice (*hCD2*^*eYFP*^) (Siegemund et al., 2015; Dress et al., 2019; Sulczewski et al., 2023). In these mice, eYFP labeling was detected in both thymic pDCs and tDC populations, whereas other thymic DC populations showed low levels of YFP (Fig. 4 F). Finally, we used *Itgax*^*Cre*^*Tcf4*^*fl/fl*^ mice, which lack TCF4 expression specifically in CD11c-expressing cells. TCF4 has been previously described as essential for pDC and tDC development (Cisse et al., 2008; Sulczewski et al., 2023). Genetic ablation of TCF4 significantly reduced the numbers of both thymic pDC and tDC populations (Fig. 4 G), confirming their shared developmental origin.

Overall, the distinct transcriptional profile of CX3CR1^+^ DC2, their shared origin with pDCs, and their developmental dependence on TCF4 expression clearly identify these cells as previously unrecognized thymic tDCs.

### tDCs represent transendothelial cells

**Figure S4.**
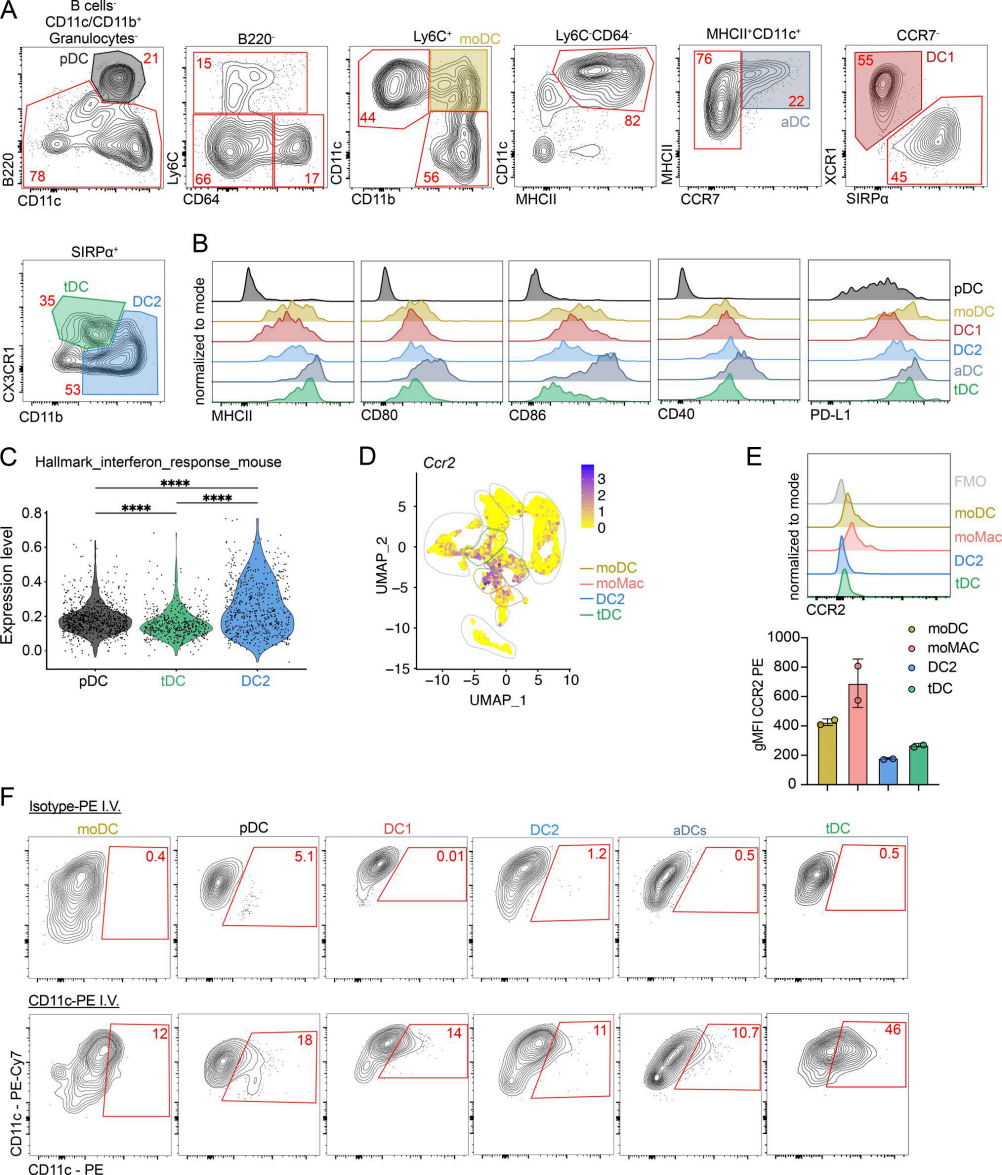
**tDCs represent transendothelial cells. (A)** Representative flow cytometry gating strategy of thymic myeloid cells. Cells were pre-gated as shown in Fig. S1 D. The gating strategy identifies pDCs (B220^+^CD11c^+^), moDCs (B220^−^Ly6C^+^CD11b^+^CD11c^+^), aDCs (CD11c^+^MHCII^+^CCR7^+^), DC1 (CD11c^+^MHCII^+^CCR7^−^XCR1^+^), DC2 (CD11c^+^MHCII^+^CCR7^−^SIRPα^+^CD11b^+^), and tDCs (CD11c^+^MHCII^+^CCR7^−^SIRPα^+^CD11b^low^CX3CR1^+^). **(B)** Representative flow cytometry plots showing the normalized expression of MHCII, CD80, CD86, CD40, and PD-L1 in thymic DC populations. Cells were gates as shown in A. **(C)** Violin plot displaying the normalized expression of gene associated with Hallmark IFN response mouse in clusters defined in Fig. 4 B. **(D)** Feature plot showing the normalized expression of *Ccr2* in clusters identified in Fig. 1 A. **(E)** Representative flow cytometry plot showing normalized expression of CCR2 in thymic myeloid cell populations. Graph shows gMFI of CCR2 expression by thymic myeloid cells; *n* = 2 from two independent experiments. Data are shown as mean ± SD. **(F)** Representative flow cytometry plots showing analysis of *ex vivo* anti–CD11c-PE-Cy7 and i.v. anti–CD11c-PE labeling in thymic populations of moDCs and DCs. Statistical analysis was performed by a Wilcoxon run-sum test; ****P ≤ 0.0001.

**Figure 5.**
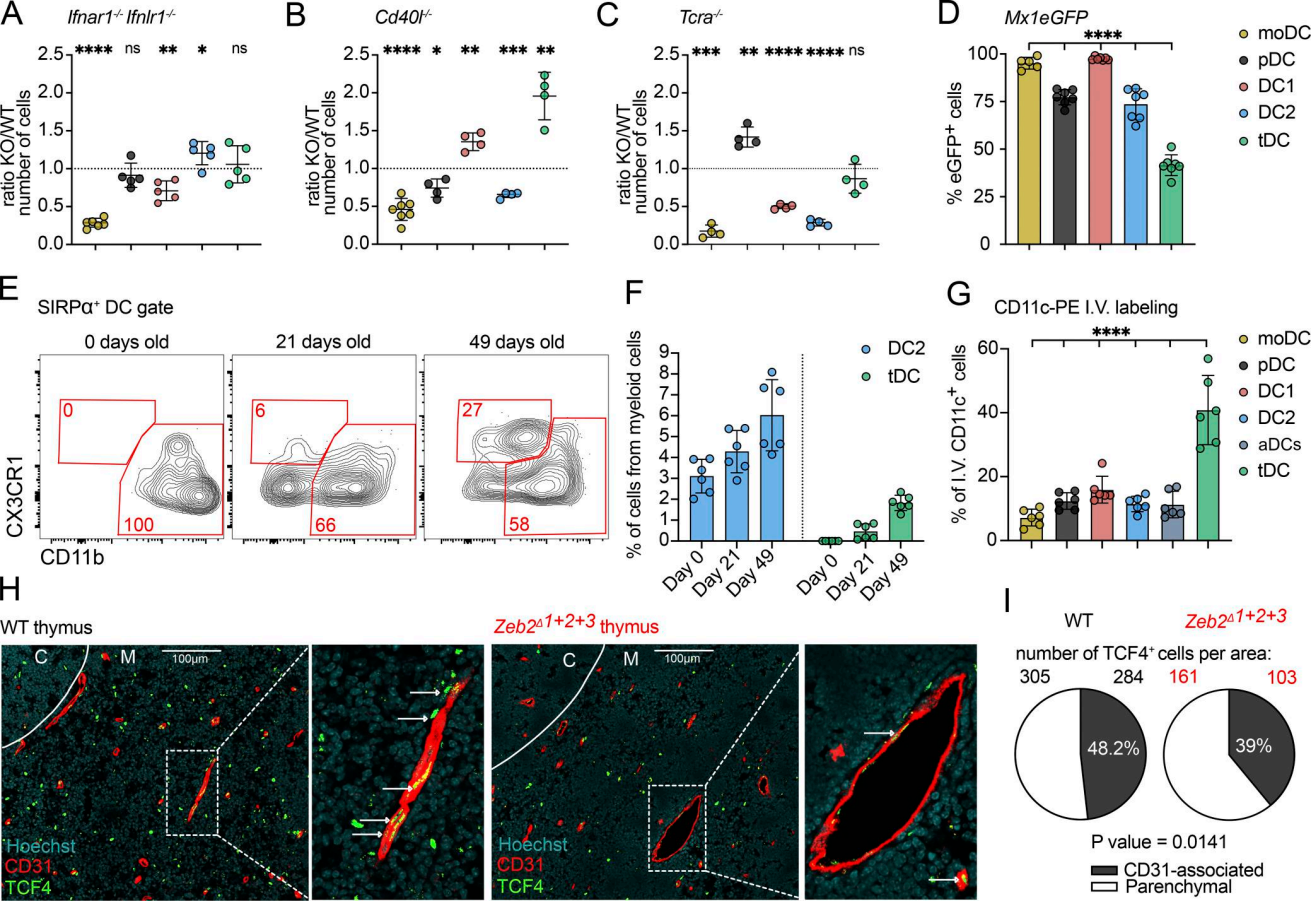
**tDCs represent transendothelial cells. (A)** Numbers of thymic moDCs and DCs (gated as in Fig. S4 A) in *Ifnar1*^*−/−*^ and *Ifnlr1*^*−/−*^ mice, shown as KO/WT ratio of cell numbers; *n* = 5–6 mice from two independent experiments. **(B)** Numbers of thymic moDC and DCs in *Cd40l*^*−/−*^ mice, shown as KO/WT ratio of cell numbers; *n* = 4–7 mice from two independent experiments. **(C)** Numbers of thymic moDC and DCs in *Tcra*^*−/−*^ mice, shown as KO/WT ratio of cell numbers; *n* = 4 mice from two independent experiments. **(D)** Frequency of eGFP^+^ thymic moDC and DCs in *Mx1*^*eGFP*^ mice; *n* = 5–7 mice from three independent experiments. **(E)** Representative flow cytometry plots showing thymic SIRPα^+^ DCs (gated as shown in Fig. S4 A) from birth (0 days) to 49-day-old mice. **(F)** Frequency of DC2 and tDC among total CD11c^+^ and CD11b^+^ cells in the thymus of C57BL/6 mice from birth (0 days old) to 49-day-old mice; *n* = 6 mice from two independent experiments. **(G)** Frequency of labeled thymic moDC and DCs by i.v. administration of anti–CD11c-PE antibody. Mice were euthanized 2 min after administration; *n* = 6 mice per three independent experiments. The cells were gated as shown in Fig. S4 F. **(H)** Representative confocal microscopy images comparing the localization of TCF4^+^ cells in the thymus of WT C57BL/6 and *Zeb2*^*Δ1+2+3*^ mice. The association with thymic microvessels was assessed by colocalization of TCF4^+^ cells with CD31^+^ positivity. Medullary region was identified by Hoechst staining. Scale bars represent 100 μm. **(I)** Numbers of free and CD31-associated TCF4^+^ cells in the specific thymus area of WT C57BL/6 and *Zeb2*^*Δ1+2+3*^ mice. Data are shown as mean ± SD. Statistical analysis was performed by a one-sample *t* test and Wilcoxon test with a theoretical mean of 1 (A–C), one-way ANOVA with multiple comparisons analysis (D and G), and a two-sided Fisher’s exact test (I); *P ≤ 0.05, **P ≤ 0.01, ***P ≤ 0.001, ****P ≤ 0.0001, and n.s., not significant.

Thymic tDCs represent a mature population of DCs, expressing high expression of MHCII, comparable with that of CCR7^+^ aDCs (Fig. S4, A and B). Additionally, tDCs express various co-stimulatory molecules, albeit at lower levels than conventional aDCs (Fig. S4 B). This suggests that, while thymic tDCs are activated cells, their activation state remains distinct from conventional thymic DCs. To investigate their mode of activation, we quantified the numbers of thymic tDCs in *Ifnar1*^*−/−*^*Ifnlr1*^*−/−*^, *Cd40l*^*−/−*^, and *Tcra*^*−/−*^ mice, models in which the thymic maturation of various APCs is altered as described above (Fig. 5, A–C). Interestingly, the number of tDCs was not reduced in any of these models. This suggests that tDC do not require the same local signals (IFN and CD40L) that activate other thymic DC. Furthermore, thymic tDCs exhibited lover levels of IFN response and decreased Mx1^eGFP^ expression compared with other thymic APC, suggesting their limited IFN sensing in the thymus (Fig. S4 C and Fig. 5 D). This finding raises the possibility that tDCs may correspond to a previously described population of CX3CR1^+^ DCs capable of migrating into the thymus from peripheral tissues. Indeed, prior studies indicated that thymic CX3CR1^+^ DCs increase in number after weaning (∼21 days of life), yet their origin and characteristics were not addressed (Zegarra-Ruiz et al., 2021). Our transcriptional and lineage-tracing data showed that thymic CX3CR1^+^ DC can be comprised of moDC, moMac, and tDCs (Fig. 2, C and F; and Fig. 4, C and F). Notably, monocytes and macrophages are abundant early in life but decline with age (Fig. 1 D), while tDCs are completely absent at birth, begin to appear around day 21 and increase over the first 7 wk of life (Fig. 5, E and F). Additionally, the migration of SIRPα^+^ DCs into the thymus has previously been linked to CCR2 signaling (Baba et al., 2009). Although *Ccr2* mRNA expression is detectable across all SIRPα^+^ DCs subsets (Fig. S4 D), CCR2 protein expression is restricted to moDC and moMac populations (Fig. S4 E). This suggests that the immigration of thymic tDCs occurs independently of CCR2 signaling.

Previous work identified a population of transendothelial DC residing near microvessels in the thymic medulla as a circulating migratory DC population that brings blood-borne antigens into the thymus (Vollmann et al., 2021). These cells exhibited conventional DC2 characteristics and expressed CX3CR1 (Vollmann et al., 2021). To determine whether thymic tDCs correspond to this transendothelial population, we i.v. injected mice with anti–CD11c-PE mAb, euthanized them 2 min after injection, and analyzed the thymic DC pool via flow cytometry. The results showed that thymic tDCs bound the anti-CD11c mAb much more efficiently than other thymic DC subsets, with over 40% of these cells stained by i.v. labeling (Fig. 5 G and Fig. S4 F). This finding suggests that a substantial portion of tDCs are exposed to the bloodstream. Furthermore, all i.v. labeled tDCs also displayed positivity when stained *ex vivo* with anti–CD11C-PE-Cy7 (Fig. S4 F), further confirming their transendothelial phenotype (Vollmann et al., 2021).

**Figure S5.**
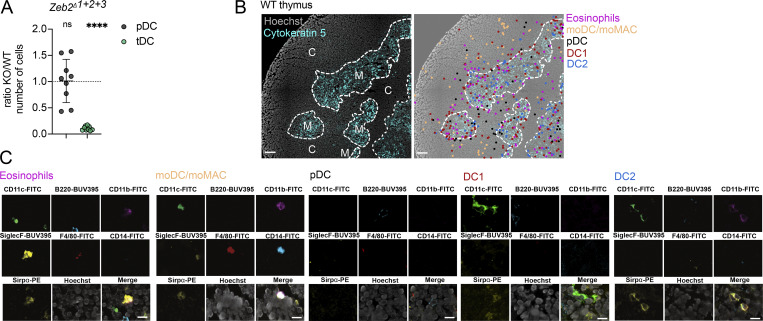
**Intrathymic localization of thymic myeloid cells. (A)** Numbers of thymic pDC and tDC in *Zeb2*^*Δ1+2+3*^ mice. Cells were gated as shown in Fig. S4 A. Data are shown as KO/WT ratio of cell numbers; *n* = 9 mice from three independent experiments. Data are shown as mean ± SD. **(B)** Representative microscopy images using Canopy CellScape showing the intrathymic localization of eosinophils, monocyte-derived cells (moDC and moMac), pDC, DC1, and DC2. Medullary region was identified by Cytokeratin 5 staining. Scale bars represent 100 μm. **(C)** Representative microscopic images using Canopy CellScape showing expression of specific markers enabling the identification of individual thymic myeloid cell subsets. The cell specific markers enable to identify: eosinophils (CD11c^+^SiglecF^+^CD11b^+^SIRPα^+^), moDC/moMac (CD11c^+^CD11b^+^SIRPα^+^F4/80^+^ CD14^+^), pDC (B220^+^SIRPα^+^), DC1 (CD11c^+^CD11b^+^SIRPα^−^), and DC2 (CD11c^+^CD11b^+^SIRPα^+^). Scale bars represent 10 μm. Statistical analysis was performed by one-sample *t* test and Wilcoxon test with a theoretical mean of 1; ****P ≤ 0.0001, and n.s., not significant.

To test if thymic tDCs are positioned near microvessels, we performed confocal microscopy on frozen thymic sections stained with antibodies against TCF4 and the endothelial marker CD31 (Fig. 5 H, left panel). Notably, nearly 50% of TCF4^+^ cells (which includes pDC and tDC) were in close contact with CD31^+^ endothelial cells, indicating their presence in the perivascular space. The remaining half of the TCF4^+^ cells were located within the thymic parenchyma (Fig. 5, H and I), aligning with our i.v. labeling data, where ∼40% of tDCs were labeled (Fig. 5 G). To distinguish tDC from pDC, we quantified TCF4^+^ cells in *Zeb2*^*Δ1+2+3*^ mice, which lack tDCs while retaining pDCs (Fig. S5 A). Interestingly, perivascular (CD31-associated) were strongly reduced in *Zeb2*^*Δ1+2+3*^ mice (Fig. 5, H and I). This suggests that the majority of CD31-associated TCF4^+^ cells correspond to tDCs. Whereas tDCs are predominantly localized in thymic medullary near microvessels, other SIRPα^+^ cell populations display distinct spatial distributions. Classical DC2 are found predominantly throughout the medullary region and at the cortico-medullary junctions, while moDC and moMac are primarily localized in the thymic cortex. Interestingly, pDCs are dispersed across both the cortical and medullary region (Fig. S5, B and C).

Together, these findings demonstrate the previously unappreciated finding that thymus-immigrating DCs are tDCs of shared developmental origin with pDC.

## Discussion

Central tolerance is a crucial mechanism that prevents autoimmunity by eliminating self-reactive T cells during thymic selection. The magnitude of central tolerance is shaped by the diversity of self-p:MHC that developing thymocytes encounter on thymic APCs (Klein and Petrozziello, 2024). The self-peptidome displayed by different thymic APC subsets varies significantly between cell types and is influenced by factors, such as developmental origin, maturation, and activation state, and key molecules that drive APC differentiation within the thymus or peripheral tissues (Spencer et al., 2015; Kim et al., 2025; Canesso et al., 2024). Notably, thymic hematopoietic APCs exhibit a high degree of heterogeneity, the extent and functional implications of which remain incompletely understood. Here, we employed high-resolution scRNA-seq, complemented by various lineage-tracing and lineage-defining mouse models, to characterize the origin and lineage identity of thymic myeloid cells. In particular, we explored the internal heterogeneity within SIRPα^+^ DCs and found that what was previously described as “cDC2” is composed of four developmentally distinct lineages. These include moDC and moMacs, conventional DC2 with their activated counterparts (aDC2), and tDCs, which share a developmental origin with pDCs.

In this study, we identified four major populations of *Mafb*-expressing cells of monocyte/macrophage origin (Wu et al., 2016) (Fig. 2, A–C). Phenotypically, these two lineages can be distinguished based on the expression of Ly6C, CD64, and MERTK. Monocytes express high levels of Ly6C, whereas macrophages are Ly6C-negative but express high levels of CD64 and MERTK. Notably, both populations contain thymus-specific subsets that upregulate CD11c, MHCII, and co-stimulatory molecules (Fig. 2, D and E; and Fig. S2 C). Moreover, the presence of these CD11c^+^MHCII^+^ monocytes and macrophages in the thymus is highly dependent on type I and type III IFN signaling (Fig. 2 J). These characteristics led to the hypothesis that these cells represent activated subsets of monocytes and macrophages (Ashby et al., 2024). However, the fate-mapping experiments using the *Ms4a3*^*Cre*^ROSA26^tdTomato^ mouse model clearly attributed both CD11c^+^MHCII^+^ monocytes (moDC) and CD11c^+^MHCII^+^ macrophages (moMac) to the monocyte lineage (Fig. 2 F). This finding suggests that thymic macrophage subsets represent distinct lineage identities rather than different activation states (Liu et al., 2019). This aligns with a previous study describing thymic macrophage heterogeneity, which identified two major populations: TIM4^+^ embryonic-derived tissue-resident macrophages and hematopoietic stem cell–derived CX3CR1^+^ macrophages (Zhou et al., 2022). We believe that these correspond to macrophages and moMacs described in this study. Additionally, we hypothesize that these subsets also differ functionally, as MHCII^Low^ TIM4^+^ macrophages exhibit limited antigen-presenting capacity, whereas moMacs, under the influence of IFNs, upregulate MHCII and co-stimulatory molecules, rendering them highly specialized thymic APCs (Zhou et al., 2022). Interestingly, the phenotypic characterization of moDCs and moMacs resembles the recently described population of conventional DC-type 3 (DC3). DC3s arise from Ly6C^+^ MDP-derived progenitor, require FLT3 signaling, and express conventional DC transcription factor and markers, such as *Zbtb46* and *Dpp4* (CD26), while simultaneously upregulating monocyte-associated markers, including CX3CR1 and CD16/32 (Liu et al., 2023; Rodrigues et al., 2024). In contrast, thymic moDCs and moMacs lack expression of F*lt3* and CD26 and display high levels of recombination in *Ms4a3*^*Cre*^ROSA26^tdTomato^ mouse model (Fig. S1, B and E; and Fig. 2, E and F). These findings suggest that thymic moDC and moMac are distinct from splenic DC3s and instead represent populations of homeostatically activated, monocyte-derived cells. The identification of moDCs and moMacs that resemble conventional DC2 through the upregulation of CD11c, MHCII, and co-stimulatory molecules provides valuable insight into the thymic APC subsets that are functionally equipped to induce T cell tolerance. The detailed characterization and lineage identity of these monocyte-derived thymic APCs, presented in this study, will facilitate further research exploring the specific role of these subtypes in T cell clonal deletion and/or Treg selection.

The thymic DC pool contains homeostatically activated subsets characterized by the upregulation of CCR7, MHCII, and co-stimulatory molecules (Park et al., 2020; Oh et al., 2018; Ardouin et al., 2016). DC activation is a process in which DCs transition from immature antigen-capturing cells to fully activated APCs capable of highly efficient antigen presentation (Guermonprez et al., 2002; Maier et al., 2020). Here, we show that thymic aDCs represent a continuum of cells spanning from early activated populations, which express both CCR7 and DC1 or DC2 lineage-defining molecules, such as XCR1 or SIRPα, to fully matured late aDCs that substantially downregulate transcriptional and protein characteristics of their respective DC lineage. Notably, the DC1 population exhibited a more pronounced activation effect, as the CCR7^+^XCR1^−^ SIRPα^−^ cells predominantly represent aDC1 (Fig. 3, D and E; and Fig. S3, E and F). Our study provides evidence that both thymic aDC populations are activated subtypes of DC1 and DC2, respectively, as they show clear dependence on either the DC1 lineage-defining *Batf3*^*−/−*^ mouse model or the DC2-depleting *Zeb2*^*Δ1+2+3*^ mouse (Fig. 3 F). Interestingly, despite their distinct origins, aDC1 and aDC2 cells share highly similar transcriptional profiles, suggesting that they undergo a universal DC maturation program, ultimately leading to the formation of functionally convergent APCs within the thymus (Ardouin et al., 2016; Breed et al., 2022). Notably, the depletion of one DC subset is compensated by an increase in the other, preserving the total number of thymic DCs in both *Batf3*^*−/−*^ and *Zeb2*^*Δ1+2+*^ mice (Fig. 3 F) (Hildner et al., 2008; Liu et al., 2022). We hypothesize that this mutual compensation between the thymic DC1 and DC2 lineages is driven by the availability of vacant niches that would normally be occupied by either subset. This dynamic adjustment presents a significant challenge in studying the distinct functions of DC1 and DC2 cells in central tolerance, as the remaining subset may occupy a similar thymic microenvironment, facilitating their specific role in T cells selection.

As described previously, several thymic microenvironmental signals regulate APC maturation within the thymus (Ashby et al., 2024; Oh et al., 2018; Breed et al., 2022). Here, we show that the activation of moDC, moMac, and DC1 is highly regulated by type I and type III IFN signaling, whereas the maturation of DC2 relies on CD40^−^CD40L signaling. Notably, the absence of SP thymocytes in *Tcra*^*−/−*^ mice leads to a marked reduction in all activated thymic APC subtypes, including moDC, moMac, aDC1, and aDC2 (Fig. 2 J and Fig. 3 G). This finding aligns with previous study highlighting the importance of CD4SP thymocytes and CD40 signaling in thymic DC2 maturation, with a lesser role in DC1 maturation (Oh et al., 2018). These results underscore the role of SP thymocytes in thymic APC maturation, either by providing CD40L or by promoting thymic IFN production. However, the direct effect of SP thymocytes on thymic APC maturation remains unclear, as the presence of SP T cells and the ablation of CD40L signaling also affect medullary thymic epithelial cells, which are key producers of type I and type III IFN, as well as other cytokines and chemokines (Akiyama et al., 2008; Ashby et al., 2024). Thus, it remains to be determined whether SP T cells directly signal to thymic DCs to promote their activation or whether they modulate the overall thymic microenvironment, leading to secondary effects on APC maturation.

Historically, DC1 were described as thymus resident, originating from intrathymic differentiation, whereas DC2 were thought to migrate from the periphery as fully differentiated cells (Porritt et al., 2003; Bonasio et al., 2006). However, previous studies using mouse parabiosis or photoconvertible mouse models suggested that only a minority of DC2 cells possess the ability to migrate into the thymus (Breed et al., 2022; Zegarra-Ruiz et al., 2021). It has been observed that pDC can enter the thymus in a CCR9-dependent fashion and present model antigens to developing T cells (Hadeiba et al., 2012). More recent research identified a population of transendothelial DC, localized near microvessels, enabling the transfer and presentation of blood-borne antigens to developing T cells. The transendothelial positioning of these cells depends on CX3CR1 signaling (Vollmann et al., 2021). Additionally, a population of CX3CR1^+^ DCs has been observed to migrate into the thymus early in life, inducing the expansion of microbiota-specific T cells (Zegarra-Ruiz et al., 2021). Here, we showed that the thymus accommodates a unique population of tDCs, phenotypically defined as TCF4^+^CX3CR1^+^CD14^+^SiglecH^−^CD11b^Low^ cells, which share a developmental origin with pDCs (Fig. 4, C–G). These cells efficiently bound i.v. injected CD11c-PE antibody, were localized near thymic microvessels, and were present in the thymus only at later time points after the mouse weaning period (Fig. 5, E–G). These characteristics clearly suggest that the described tDCs represent thymus-immigrating and transendothelial DCs. Furthermore, based on their characteristics—such as their shared origin with pDCs and higher MHCII expression compared with thymic pDCs—we hypothesized that they may also represent the previously described thymus-immigrating “pDCs” responsible for model blood-borne antigen presentation (Hadeiba et al., 2012). However, further investigation is required to confirm this hypothesis.

Recent studies have shown that classical DC2s in the spleen can be subdivided into two major subsets, DC2a and DC2b, which represent developmentally distinct branches of DC2 lineage (Brown et al., 2019; Rodrigues et al., 2024; Zhu et al., 2025). However, this splenic DC2 subclassification and nomenclature cannot be directly applied to thymic cells as the defining makers of DC2a and DC2b, such as *Esam*, *Dtx1*, and *Clec4a3*, are not detectable in thymic DC2s. Therefore, we chose to use the nomenclature DC2 and tDC, which more accurately reflects the phenotypic and developmental characteristics of these populations in the thymus.

The exact role of DC2 populations in T cell selection remains unresolved, primarily due to the lack of comprehensive genetic tools that allow specific targeting of DC2. However, several studies utilizing partial DC2 depletion or model antigen presentation restricted to DC2 subsets suggest that thymic DC2 populations are more specialized for T cell clonal deletion rather than Treg selection (Breed et al., 2022; Bonasio et al., 2006; Vollmann et al., 2021). Previously, we demonstrated that a substantial proportion of DC2 cells express CD301b lectin and that ablation of these cells significantly impairs the CD4^+^SP deletion (Breed et al., 2022). Notably, both DC2 and tDCs subpopulations express CD301b (Fig. 4 C), making it difficult to distinguish functional differences between these populations. Here, we provide evidence that the thymic DC2 lineage comprises two major, developmentally unrelated populations, allowing for the specific targeting of one subset to clarify its unique role in thymic T cell selection.

## Materials and methods

### Mice

5- to 8-wk-old (unless otherwise stated) male and female age-matched mice were used for experiments. Mice were housed in a specific pathogen–free facility under a 12-h light:dark cycle at 22 ± 2°C. C57BL/6J-*Ms4a3*^*em2(cre)Fgnx*^/J (*Ms4a3*^*Cre*^), B6.Cg-*Gt(ROSA)26Sor*^*tm14(CAG-tdTomato)Hze*^/J (*ROSA26*^*tdTomato*^), B6.129S2-*Ifnar1*^*tm1Agt*^/Mmjax (*Ifnar1*^*−/−*^), B6.129S2-*Cd40lg*^*tm1Imx*^/J (*Cd40l*^*−/−*^), B6.129S2-*Tcra*^*tm1Mom*^/J (*Tcra*^*−/−*^), B6(129S4)-*Xcr1*^*tm1.1(cre)Kmm*^/J (*Xcr1*^*Cre*^), B6.129X1-*Gt(ROSA)26Sor*^*tm1(EYFP)Cos*^/J (*ROSA26*^*eYFP*^), B6.129S(C)-*Batf3*^*tm1Kmm*^/J (*Batf3*^*−/−*^), and B6.Cg-Tg(Itgax-cre)1-1Reiz/J (*Itgax*^*Cre*^) were purchased from Jackson Laboratories. *Zeb2*^*Δ1+2+3*^ mice (Liu et al., 2022) were kindly provided by K. Murphy (Washington University in St. Louis, St. Louis, MO, USA). *Ifnlr1*^*tm1.2Svko*^ (*Ifnlr1*^*−/−*^) mice (Lin et al., 2016) were kindly provided by S.V. Kotenko (Rutgers New Jersey Medical School, Newark, NJ, USA). B6.Cg-Mx1^*tm1.1Agsa*^/J (*Mx1*^*eGFP*^) mice (Uccellini and García-Sastre, 2018) were kindly provided by A. García-Sastre (Icahn School of Medicine at Mount Sinai, New York, NY, USA). *Tcf4*^*fl/fl*^ mice (Cisse et al., 2008) were kindly provided by B. Reizis (New York University, New York, NY, USA). B6.Cg-Tg(CD2-icre)4Kio/J (*hCD2*^*iCre*^) were kindly provided by J. Idoyaga (University of California San Diego, San Diego, CA, USA). All animal experiments were approved by the Institutional Animal Care and Use Committee of University of Minnesota.

### Cell isolation and flow cytometry

Thymic and splenic myeloid cells and B cells were isolated using Collagenase D (1 mg/ml; Roche) dissolved in Hank’s balanced salt solution containing 2% FBS, 10 mM HEPES, and Ca^2+^Mg^2+^ ions. Tissues were finely minced in 900 μl of Collagenase D solution and incubated at 37°C for 15 min. The suspension was then pipetted up and down several times before undergoing a second incubation at 37°C for 20 min. After enzymatic digestion, the cell suspension was passed through 70-μm cell strainers, and the reaction was stopped by adding PBS with 2% FBS and 2 mM EDTA. Red blood cells from the thymus and spleen were lysed using ACK Lysis buffer (prepared in-house). For surface staining, cells were first incubated with an Fc block (anti-CD16/CD32; 2.4G2; Tonbo Biosciences) for 15 min at 4°C. This was followed by a 30-min incubation at 37°C with an anti-CCR7 antibody (4B12; Thermo Fisher Scientific). After washing, cells were further stained for 30 min at 4°C with the indicated surface antibodies. For intracellular TCF4 staining, cells were fixed with FoxP3 Transcription Factor Fix/Perm buffer (Thermo Fisher Scientific) for 2 h and stained for 30 min in 1× Permeabilization buffer (Thermo Fisher Scientific) at 4°C. Samples were acquired with a BD Fortessa X-20, BD Fortessa H1770, or Cytec Aurora U1405 and analyzed with FlowJo v.10.10 (FlowJO LLC).

### Antibodies

Antibodies purchased from BioLegend were the following: CD11c (N418), CD11b (M1/70), XCR1 (ZET), CX3CR1 (SA011F11), CD64 (X54-5/7.1), I-A/I-E (M5/114.15.2), CD14 (Sa14-2), CD172a (P84), CD88 (20/70), CD274 (10F.9G2), CD86 (A17199A), CD26 (H194-112), MERTK (2B10C42), VCAM-1 (429), TIM4 (RMT4-54), CCR2 (SA203G11), and CD63 (NVG-2). Antibodies purchased from BD Biosciences were the following: Siglec-F (E50-2440), Ly-6G (1AB), CD45R/B220 (RA3-6B2), Siglec-H (440c), CD197 (4B12), and CD40 (3/23). Antibodies purchased from Tonbo Biosciences were the following: CD80 (16-10A1) and F4/80 (BM8.1). Antibodies purchased from Thermo Fisher Scientific were the following: Ly-6C (HK1.40). Antibodies purchased from Abcam were the following: TCF-4 (NCI-R159-6).

### scRNA-seq and analysis

Sequencing and initial analysis were done at the University of Minnesota Genomics Center of the University of Minnesota as described previously (Breed et al., 2022). Thymic myeloid cells were isolated as described above, and cells were MACS enriched for CD90.2^−^ cells to deplete lymphocytes. CD11c/CD11b^+^ cells were FACS sorted and captured using the 10X Genomics 3′Single Cell v.3 chemistry platform and sequenced in a NovaSeq instrument. Prior to sequencing the quality control was assessed by Illumina-basicQC. Raw count data were loaded into R (v.4.4.1) and analyzed with the Seurat R package (v.5.1.0). The dataset originally contained cells from multiple conditions identified by hashtag oligonucleotide (HTO) labeling. The Seurat function “HTODemux” was used to identify “doublet” cells. After filtering out doublets, only C57BL/6J WT cells were selected for subsequent analysis. The mRNA expression data were then normalized using a log normalization method, where gene expression counts were normalized and scaled to correct for differences in sequencing depth and technical noise. To identify the most informative genes for downstream analysis, the “FindVariableFeatures” function in Seurat was used to select 2,000 highly variable genes. These features were then used for subsequent analyses, ensuring robust identification of cell clusters and states. Dimensionality reduction was performed using the “RunPCA” function. The top principal components were used as input for “RunUMAP,” which generated a two-dimensional visualization of the data based on the Uniform Manifold Approximation and Projection (UMAP) algorithm. Cell clustering was performed using the “FindClusters” function in Seurat, which applies a shared nearest neighbor–based clustering approach. To visualize the clustering, results the “DimPlot” function was used, which represents cells in UMAP space and colors them according to their assigned clusters. Gene expression patterns across clusters were visualized using “FeaturePlot,” which overlays expression levels of individual genes onto the UMAP projection. To identify differentially expressed genes (DEGs) between clusters, we applied the “FindMarkers” function, which performs statistical testing (Wilcoxon’s rank-sum test) to detect genes with significant expression differences between cell populations. These analyses and visualizations were conducted using R packages, including Seurat (v.5.1.0), ggplot2 (v.3.5.1), dplyr (v.1.1.4), and SeuratObject (v.5.0.2).

### Intravascular labeling

Intravascular labeling of thymic cells was done as described previously (Vollmann et al., 2021). Cells were labeled by i.v. injection of 1 μg of PE-conjugated anti-CD11c mAb (clone N418). Mice were euthanized by CO_2_ asphyxiation followed by cervical dislocation 2 min after mAb injection. Isotype-PE antibody i.v. injection was used as control. Thymic myeloid cells were then isolated and analyzed as described above.

### Immunofluorescence

Thymi were from C57BL/6J WT and were fixed in Cytofix/Cytoperm (BD Biosciences) at 4°C for 24 h, followed by two washes in PBS. The tissues were then incubated in 30% sucrose at 4°C for 24 h for cryoprotection. Afterward, the thymi were embedded in OCT compound (Sakura Finetek), frozen in the vapor phase of liquid nitrogen, and stored at −80°C until further processing. For analysis, frozen sections were dried overnight, rehydrated in PBS for 5 min, and blocked at room temperature for 60 min in PBS containing 0.3% Triton X-100, 1% BSA, and mouse Fc block. Sections were stained with primary antibodies TCF4 and CD31 (MEC13.3) overnight at 4°C. Sections were washed three times with PBS and stained with Hoechst 33342 (Thermo Fisher Scientific) for 5 min at room temperature. Sections were washed and mounted in ProLong Gold Antifade. Images were acquired using a Stellaris 8 confocal microscope (Leica Microsystems) and analyzed using Fiji and QuPath. The numbers of TCF4^+^ cells were calculated manually within the area of 1.7 mm^2^.

### Canopy CellScape microscopy

Thymic sections were prepared as described above. Following blocking, samples were washed three times with PBS and then placed into the CellScape Tissue Chip. Samples were installed onto the CellScape Canopy and imaged as per the manufacturer’s protocol with the following panel: Cytokeratin 5 (EP1601Y; Abcam), CD11c (N418; Thermo Fisher Scientific), B220 (RA3-6B2; BD Biosciences), CD11b (5C6; BioLegend), SiglecF (E50-2440; BD Biosciences), F4/80 (BM8; Thermo Fisher Scientific), CD14 (Sa14-2; BioLegend), SIRPα (P84; BioLegend), and Hoechst (Thermo Fisher Scientific). Cytokeratin 5 staining was used to discriminate cortical and medullary regions of the thymus. Generated OME-Tiff files were analyzed and annotated manually with QuPath software. The figures were generated using QuickFigures in Fiji, allowing the visualization of larger thymus area.

### Statistical analysis

For comparison of three or more datasets, ordinary ANOVA with multiple comparisons test was used. One-sample *t* test and Wilcoxon test were used to perform single-column statistics. A two-sided Fisher’s exact test was used to analyze the differences in cell localization in microscopic images. Wilcoxon’s rank-sum test was used to identify DEGs in scRNA-seq. P < 0.05 was considered significant. Sample size, experimental replicates, and additional details are provided in the figure legends. Statistical analyses were performed using GraphPad Prism 9.0.

### Online supplemental material

Fig. S1 shows unfiltered data from scRNA-seq analysis of thymic CD11c/CD11b^+^ cells and provide supporting data on thymic myeloid cell gating strategy. Fig. S2 provides supporting data on the heterogeneity and regulation of thymic monocyte and macrophages populations. Fig. S3 shows clustering of thymic DC populations from scRNA-seq analysis, provides the analysis of thymic CCR7^+^ pDCs, and provides supporting data on thymic aDCs maturation. Fig. S4 shows flow cytometry gating strategy for thymic DCs and provide supporting data on thymic tDC migration. Fig. S5 shows dependence on thymic tDC and pDC on *Zeb2* enhancer and provide data describing the intrathymic localization of myeloid cells.

## Data Availability

scRNA-seq data are available in the NCBI’s GEO (https://www.ncbi.nlm.nih.gov/geo/) under accession number GSE198247. The main data supporting the findings of the present study are available in the article’s supplementary figures. Data are available from the corresponding authors upon request.
